# Epstein-Barr virus perpetuates B cell germinal center dynamics and generation of autoimmune-associated phenotypes *in vitro*

**DOI:** 10.3389/fimmu.2022.1001145

**Published:** 2022-09-28

**Authors:** Elliott D. SoRelle, Nicolás M. Reinoso-Vizcaino, Gillian Q. Horn, Micah A. Luftig

**Affiliations:** ^1^ Department of Molecular Genetics & Microbiology, Duke University, Durham, NC, United States; ^2^ Department of Biostatistics & Bioinformatics, Duke University, Durham, NC, United States; ^3^ Department of Immunology, Duke University, Durham, NC, United States

**Keywords:** Epstein-Barr virus, B cell, germinal center, single-cell, lymphoblastoid cells, autoimmunity, chronic infection, atypical memory B cells

## Abstract

Human B cells encompass functionally diverse lineages and phenotypic states that contribute to protective as well as pathogenic responses. Epstein-Barr virus (EBV) provides a unique lens for studying heterogeneous B cell responses, given its adaptation to manipulate intrinsic cell programming. EBV promotes the activation, proliferation, and eventual outgrowth of host B cells as immortalized lymphoblastoid cell lines (LCLs) *in vitro*, which provide a foundational model of viral latency and lymphomagenesis. Although cellular responses and outcomes of infection can vary significantly within populations, investigations that capture genome-wide perspectives of this variation at single-cell resolution are in nascent stages. We have recently used single-cell approaches to identify EBV-mediated B cell heterogeneity in *de novo* infection and within LCLs, underscoring the dynamic and complex qualities of latent infection rather than a singular, static infection state. Here, we expand upon these findings with functional characterizations of EBV-induced dynamic phenotypes that mimic B cell immune responses. We found that distinct subpopulations isolated from LCLs could completely reconstitute the full phenotypic spectrum of their parental lines. In conjunction with conserved patterns of cell state diversity identified within scRNA-seq data, these data support a model in which EBV continuously drives recurrent B cell entry, progression through, and egress from the Germinal Center (GC) reaction. This “perpetual GC” also generates tangent cell fate trajectories including terminal plasmablast differentiation, which constitutes a replicative cul-de-sac for EBV from which lytic reactivation provides escape. Furthermore, we found that both established EBV latency and *de novo* infection support the development of cells with features of atypical memory B cells, which have been broadly associated with autoimmune disorders. Treatment of LCLs with TLR7 agonist or IL-21 was sufficient to generate an increased frequency of IgD^-^/CD27^-^/CD23^-^/CD38^+^/CD138^+^ plasmablasts. Separately, *de novo* EBV infection led to the development of CXCR3^+^/CD11c^+^/FCRL4^+^ B cells within days, providing evidence for possible T cell-independent origins of a recently described EBV-associated neuroinvasive CXCR3^+^ B cell subset in patients with multiple sclerosis. Collectively, this work reveals unexpected virus-driven complexity across infected cell populations and highlights potential roles of EBV in mediating or priming foundational aspects of virus-associated immune cell dysfunction in disease.

## Introduction

The widespread utilization of single-cell RNA sequencing (scRNA-seq) has generated new high-dimensional perspectives on the diversity of the human B cell repertoire. Recent scRNA-seq studies have revealed that extensive heterogeneity and dynamic complexity are core aspects of B cell development and clonal selection (1–4), tissue-specific distributions (5), dysregulation in lymphoma (6–9), pathogenicity in autoimmune disorders (10–12), and responses to antigen (13, 14) and infection (15, 16). Separately, advances in classic single-cell methods including flow cytometry and cell sorting (17) provide flexible workflows to validate and isolate cell subsets identified from scRNA-seq experiments and support subsequent investigations. Used in conjunction, this battery of techniques supports genome-wide profiling and niche-specific functional studies of cell behaviors and responses to stimuli. Each of these capabilities are essential to resolve the broad landscape of B cell mediated immunity, including how it is reshaped across a spectrum of diseases to the point of pathogenic dysregulation.

Arguably, the pathogen most intricately interwoven with B cell epigenomic programming and immune responses in humans is infection with Epstein-Barr Virus (EBV), a gammaherpesvirus found in approximately 95% of adults worldwide (18). The EBV virion consists of a 172 kB dsDNA genome encapsulated by an icosahedral glycoprotein capsid with associated tegument proteins. Viral particles are transmitted between hosts through saliva, from which the virus can infect oral epithelial cells, traverse the oral mucosa, and eventually encounter host B cells resident within tonsillar lymphoid tissue (18). Binding of the viral capsid glycoprotein gp350 to an extracellular domain of cell surface-expressed CR2 (CD21) facilitates viral entry through endocytosis (19–21). Internalized virions then translocate to the nucleus, where the linear viral genome is deposited and rapidly circularizes to form an extrachromosomal episome (22, 23). Successful infection is achieved through the sequential expression of six EBV nuclear antigens (EBNAs) and two latent membrane proteins (LMPs) in distinct programs that co-opt B cell immune response dynamics (24). In the pre-latent phase, EBNA2 and EBNA-LP are expressed and co-transactivate host genes involved in cell activation and proliferation (25–29). EBNA2 further transactivates viral C promoter expression of EBNA1 (30), which tethers the viral episome to host chromatin (31), and EBNA3A-C, which have diverse roles including suppression of apoptosis and moderation of cell proliferation to avoid growth-associated DNA damage induction (32–40). The resulting stage – Latency IIb – is defined by rapid division of host B cells that resembles germinal center (GC) dark zone (DZ) proliferation conventionally induced by B Cell Receptor (BCR) cognate antigen binding (41–44). EBNA2 further transactivates the expression of viral LMP1 and LMP2A/B (45, 46), yielding the Latency III program in which all EBNAs and LMPs are expressed simultaneously. This stage of infection reflects the phenotype of GC Light Zone (LZ) B cells through LMP1-mediated stimulation of pro-survival NF-κB pathway signaling (47–50) and LMP2A-mediated evasion of T cell surveillance and mimicry of an activated BCR (51–55). Infected B cells that successfully navigate the GC reaction eventually exit as effector B cell types, with progression to memory B cells (MBCs) providing the route to a lifelong reservoir for latent EBV persistence (56–58). Alternatively, host cell GC exit and differentiation to plasmablasts constitutes a host cell fate that precludes persistent latency. To bias GC output toward MBC formation, EBNA3A and EBNA3C mediate epigenetic suppression of the host gene *PRDM1*, inhibiting plasma differentiation (59). In the event that this suppression is unsuccessful, EBV can escape terminally differentiated plasma cells through activation of lytic replication (60). The protein encoded by the *BZLF1* gene orchestrates this phase, and its expression is transactivated by the host transcription factor XBP1 expressed in antibody-secreting cells (ASCs) (61–64). Lytic reactivation from plasma cells thus leads to amplified EBV virion formation and transmission to subsequent hosts.

EBV co-evolution with host B cells has yielded intimate viral adaptations to achieve lifelong latency in a subset of MBCs within virtually every infected person. Although benign in most cases, EBV infection of this cellular niche entails widespread viral association – and often etiological involvement – with lymphoproliferative diseases (65, 66). Indeed, EBV was first isolated from biopsies of endemic Burkitt Lymphoma (eBL) from pediatric patients (67), and the virus is known to be an essential etiologic factor in eBL development (68–70). EBV is also a major contributing factor to lymphomagenesis in the context of immune suppression. Virtually all instances of Hodgkin’s Disease and Primary CNS Lymphoma (PCNSL) in HIV-infected individuals are EBV-driven (71, 72), as are 90-100% of AIDS-related Primary Effusion Lymphomas (PEL) (73) and the immunoblastic subtype Diffuse Large B Cell Lymphoma (DLBCL) (74). Likewise, the virus is a crucial driver of post-transplant lymphoproliferative disease (PTLD, a DLBCL subset) in organ transplant recipients (75), as the requisite immunosuppressive regimens can ablate T cell-mediated restraint of EBV^+^ MBC clones (76). Collectively, EBV is associated with 1.5% of all cancer cases diagnosed annually (77), including non-B cell malignancies such as Nasopharyngeal Carcinoma (NPC) (68, 78) and nearly 10% of gastric cancers (79).

Associations of EBV with numerous diseases of immune impairment and autoimmunity have also been identified. These include systemic lupus erythematosus (SLE) (80–84), rheumatoid arthritis (RA) (85–89), myasthenia gravis (MG) (90), primary Sjogren’s Syndrome (pSS) (91, 92), and co-pathogenicity in chronic HIV and *Plasmodium falciparum* infections (78, 93). Hypothesized etiologic involvement of EBV in the development of multiple sclerosis (MS) (94, 95) has also been substantiated through recent epidemiologic and mechanistic studies (96, 97). Despite the observed viral associations, the functional roles that EBV plays in these illnesses remain incompletely understood. However, it is noteworthy that pathogenic expansion of a particular MBC niche – so-called “atypical” MBCs (atMBCs) – has been identified in many of the same diseases (12, 98–106). Because single-cell methods are well-suited to identify and study specific cellular niches, such approaches may help shed light on the potential significance of EBV infection with atMBCs.

Our lab has long been interested in dissecting the distinct B cell fates that develop following EBV infection (32, 43, 107–110). Recently, we have utilized single-cell sequencing to reveal previously unknown facets of the early stages of infection (15) and an unexpected degree of heterogeneity in virus-immortalized lymphoblastoid cell lines (LCLs) (111), which provide useful *in vitro* models of EBV^+^ lymphomas. This report builds upon our previous single-cell study of LCL heterogeneity to dissect the dynamics of virus-driven B cell responses. Through the integration of time-resolved FACS experiments and informatic approaches, we construct a general model of EBV-immortalized B cell dynamics. Our data also highlight potential viral contributions to pathogenic aspects of atMBCs that have described in EBV-associated autoimmune diseases, which we further investigated using LCLs as an initial model system.

## Materials and methods

### Cell lines

Lymphoblastoid cell lines (LCLs) were generated through *de novo* infection of human peripheral blood B cells at MOI = 5 with the B95-8 strain of EBV as previously described (32). All cell lines used in this study were cultured in RPMI media supplemented with 10% fetal bovine serum (FBS) at 37°C with 5% CO_2_.

### Flow cytometry and sorting

Cells were prepared for flow cytometry experiments by standard washing and staining methods. Briefly, ~2x10 (5) cells were washed *via* centrifugation for 5 min at 300 x g and resuspended in FACS buffer (1x PBS + 2% heat-inactivated Fetal Bovine Serum, FBS) for each sample of interest. Washed cells were centrifuged again and supernatant was aspirated, after which fluorescent antibody cocktails were added for biomarker staining. After 30 min of antibody incubation in the dark at ambient temperature, stained cells were washed with excess FACS buffer (up to 2 mL), centrifuged, aspirated, and resuspended for flow cytometry and/or fraction sorting. Cytometry data in the absence of sorting were acquired on a BD FACS Canto II analyzer system, and sorting experiments were performed on an Astrios Cell Sorter. Fluorescent antibodies against CD19, IgD, CCR6, CD23 (FCER2), ICAM1, CD27, CD38, CD138 (SDC1), CXCR3, CD11c(ITGAX), FCRL4, and FCLR5 were used for the experiments described herein. These include: αCD19-PE (BioLegend Cat. #302254) and αCD19-PE/Cy7 (BioLegend Cat. #302216), αIgD-PE/Cy5 (BioLegend Cat. #348250), αCCR6-PE (BioLegend Cat. #353410), αCD23-PE/Cy7 (BioLegend Cat. #338516), αICAM1-PacBlue (BioLegend Cat. #322716), αCD27-FITC (BioLegend Cat. #356404), αCD38-APC/Cy7 (BioLegend Cat. #356616), αCD138-PerCP-Cy5.5 (BioLegend Cat. #352310), αCXCR3-APC-Fire810 (BioLegend Cat. #353762), αCD11c-PE/Cy5 (BioLegend Cat. #301610), αFCRL4-PE (BioLegend Cat. #340204), and αFCRL5-APC (BioLegend Cat. #340306).

### Single-cell RNA-seq data processing

LCL scRNA-seq data were processed as described previously (111, 112). Briefly, sequencing base calls were used to generate demultiplexed reads (fastq files) *via* cellranger mkfastq with default QC parameters (CellRanger, 10x Genomics). Reads were aligned against species-concatenated reference genome packages (hg38 + NC_007605) prepared *via* cellranger mkref. Unique Molecular Identifier (UMI) read count matrices were produced from the alignment step (cellranger count). These and other single-cell LCL datasets are publicly available *via* the NIH Gene Expression Omnibus (GEO, accession GSE158275 and GSE126321). We also incorporated analysis of scRNA-seq data from discarded human tonsils generated in our lab (GSE159674).

### Single-cell data analysis and visualization

Count matrices from LCL single-cell experiments were analyzed and visualized in R using Seurat v4 (113–115). Expression counts were normalized, variable features were identified, and the datasets were subsequently integrated through identification of anchor features (*SelectIntegrationFeatures()*, *FindIntegrationAnchors()*, and *IntegrateData()* functions in Seurat). Integrated data were QC-filtered to remove any cells with <200 unique feature RNAs, >65,000 total RNA counts, and/or >10% mitochondrial reads. Next, read data were scaled and analyzed *via* principal component analysis (PCA). Cell cycle scores were also calculated to assign mitotic phases, but cell cycle regression was not performed during the scaling step. The top PCs (n = 30) were dimensionally reduced *via* uniform manifold approximation (UMAP) (116) and unsupervised clustering was performed at several resolutions to identify phenotypes and analyze differential gene expression. To correct for read dropout while preserving biological zeros, imputation *via* adaptive low-rank approximation (ALRA) (117) was applied to integrated LCL datasets using the Seurat Wrapper function *RunALRA()*. Pseudotime trajectories were calculated using Seurat Wrappers for Monocle3 (118–120) by creating and analyzing cell datasets from processed Seurat objects (*as.cell_data_set()*, *cluster_cells()*, *learn_graph()*, and *order_cells()* functions). Learned pseudotime graphs were constructed without partitioning and rooted within identified G_0_/G_1_ phase resting memory B cell clusters.

### B cell stimulation and growth assays

To investigate EBV^+^ B cell responses to IL-21 (Peprotech, Cat. #200-21) and TLR7 agonism (R848, Resiquimod, Millipore Sigma, Cat. #SML0196), LCLs were centrifuged, washed once with PBS, resuspended in fresh media to remove any secreted cytokines, and then plated at ~200,000 cells/mL in 6- or 24-well plate formats. Cell counts and viability were assayed using a Countess III system (Invitrogen) with Trypan Blue staining (1:1 ratio). Plated cells were then treated with R848 (2 μg/mL), IL-21 (10 ng/mL), or both (R848 + IL-21), and growth and viability were assayed daily relative to control cells treated with DMSO (2 μL/mL). Cells from each treatment group were analyzed by flow cytometry prior to and after stimulation at select timepoints to evaluate changes in activation state (e.g., ICAM1, CD23 positivity) and plasma cell formation (e.g. CD38, CD138 positivity). Estimates of total plasma cells by treatment were calculated from the fraction of CD38^+^/CD138^++^ cells measured by flow and cell densities from hemocytometry (Countess III measurements). Three separate LCLs (biological replicates) were assayed for each treatment condition.

## Results

### scRNA-guided isolation of distinct B cell phenotypes from EBV^+^ lymphoblastoid cells

**Figure 1:**
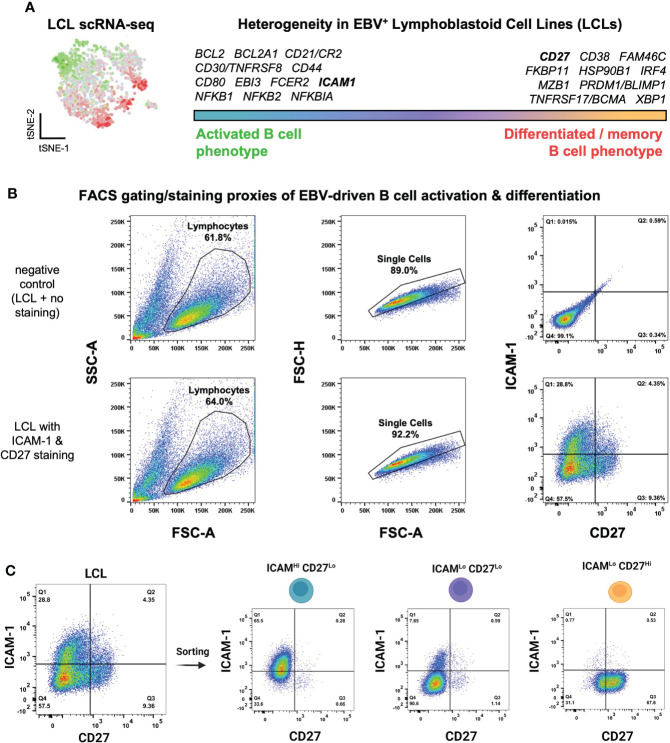
Isolation of EBV^+^ activated and differentiated B cell phenotypes identified from lymphoblastoid cell line scRNA-seq. **(A)** Top differentially expressed genes between activated and differentiated B cells from representative LCL scRNA-seq data from prior work (111). **(B)** Flow cytometry gating strategy to assess LCL heterogeneity through proxy surface marker expression. **(C)** Sorting of three ICAM/CD27 phenotypes from LCLs. ICAM-1^Hi^/CD27^Lo^ = activated B cells; ICAM-1^Lo^/CD27^Lo^ = intermediate states; ICAM-1^Lo^/CD27^Hi^ = differentiated B cells.

We previously used scRNA-seq to investigate cellular heterogeneity within EBV-immortalized lymphoblastoid cell lines (LCLs), which are *in vitro* models of B cell lymphomas of the immune suppressed (121). These data revealed an apparent continuum of EBV^+^ B cell phenotypes ranging from the early stages of cell activation and pro-survival signaling to terminal differentiation into effector memory cells or plasmablasts (**Figure 1A**). Numerous genes encoding surface-expressed proteins were differentially expressed between these activated and differentiated states. We used these findings to develop a simple FACS approach based on ICAM-1 [highly expressed on LMP-1^Hi^ activated B cells (122)] and CD27 (canonically expressed on antigen-experienced memory B cells) to further investigate LCL heterogeneity. Consistent with single-cell transcriptomic data, LCLs exhibited anticorrelated expression of ICAM-1 and CD27, with ICAM-1^Hi^/CD27^Lo^ (30-55% of cells on average) providing a proxy for GC light zone (LZ)-like activated B cells and ICAM-1^Lo^/CD27^Hi^ (5-15% of cells on average) corresponding to differentiated memory B cells (MBCs). The most frequent cell state observed by FACS was an ICAM-1^Lo^/CD27^Lo^ phenotype (40-60% of cells on average), which was interpreted as one or more transitional states (**Figure 1B**, **S1A**). Based on these observations, we used a cell sorting strategy to isolate these three major phenotypes for subsequent analysis (**Figure 1C**, **S1A**). Interestingly, the frequencies of each observed fraction were dependent upon cell density and culture conditions. When LCLs were plated at different concentrations in fresh media, both a decrease in ICAM-1^Lo^/CD27^Hi^ cells and a small (but not statistically significant) increase in the ICAM-1^Hi^/CD27^Lo^ cell frequency were observed as cell density increased. Bulk populations retained the equilibrium distribution of parental line phenotypes, provided that the cultures were maintained between 3x10 (5) - 1x10 (6) cells/mL (**Figure S1B**).

### Isolated LCL subpopulations spontaneously re-establish parental line phenotypic heterogeneity *in vitro*

**Figure 2:**
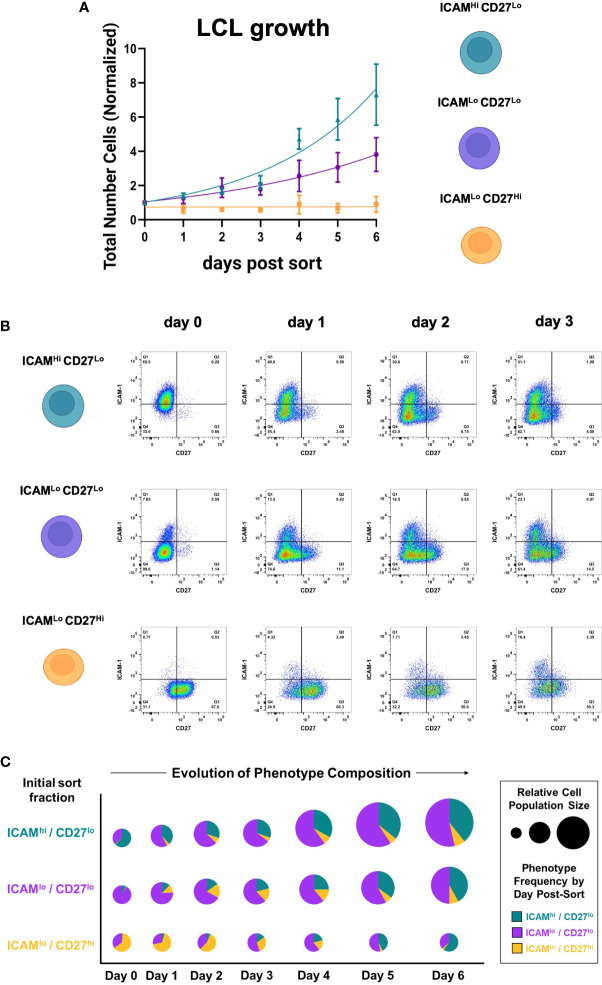
EBV^+^ activated and differentiated cell fractions spontaneously recover parental LCL heterogeneity in culture. **(A)** Growth curves from ICAM-1^Hi^/CD27^Lo^, ICAM-1^Hi^/CD27^Lo^, and ICAM-1^Hi^/CD27^Lo^ LCL fractions after sorting. Data from each day are presented as the mean number of cells normalized to initial population size at day 0 (error bars = standard deviation, n = 3 LCLs per fraction). **(B)** Representative time-resolved staining for ICAM-1 and CD27 in sorted fractions capture recovery of parental line phenotypic heterogeneity. **(C)** Fraction-resolved quantification of cell growth and phenotype distribution over six days in culture after sorting.

To better understand the growth dynamics and possible state interconversions among the three identified subpopulations (ICAM-1^Hi^/CD27^Lo^, ICAM-1^Lo^/CD27^Hi^, and ICAM-1^Lo^/CD27^Lo^), we collected cell counts and time-resolved FACS data for each fraction. Among the three fractions isolated from independent LCLs (n = 3), ICAM-1^Hi^/CD27^Lo^ cells exhibited the fastest growth, followed by ICAM-1^Lo^/CD27^Lo^ cells. The ICAM-1^Lo^/CD27^Hi^ fraction displayed minimal growth over the measurement period. However, all three phenotypes retained long-term viability in culture. These measurements demonstrated the significant growth advantage of EBV^+^ activated B cells relative to EBV^+^ differentiated B cells (**Figure 2A**, **S1C, D**). Notably, each fraction spontaneously re-established the full ICAM-1/CD27 phenotypic profile displayed by unsorted parental LCLs within several days in culture after sorting (day 0). Thus, dynamic transitions – possibly with conserved rate constants – sustain a core distribution of heterogeneous phenotypes within EBV-infected B cell populations *in vitro* (**Figure 2B, C**, **S1C, D**).

### Cyclical dynamics of GC entry, engagement, and exit are conserved across EBV^+^ LCLs

**Figure 3:**
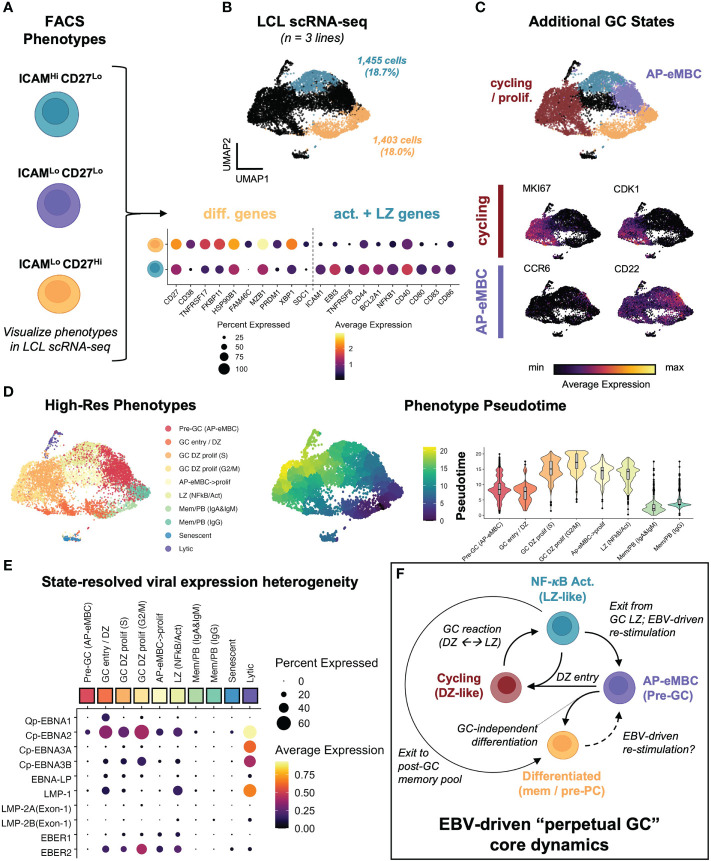
EBV perpetuates a cycle of B cell GC-like entry, engagement, and exit *in vitro*. **(A)** Dynamic phenotypes if interest from FACS experiments. **(B)** Mapping of ICAM-1^Hi^/CD27^Lo^ and ICAM-1^Lo^/CD27^Hi^ phenotypes within integrated LCL scRNA data (n = 3 LCLs). **(C)** Identification of additional cell states within LCLs. Representative marker gene UMAPs highlight states corresponding to actively cycling cells (red cluster) and pre-GC activated precursor/early MBCs (purple cluster). **(D)** Annotated clustering and pseudotime trajectory analysis of dynamic LCL states. Pseudotime scores were calculated from graphs initialized in resting MBCs (ICAM-1^Lo^/CD27^Hi^) and are presented as UMAP representations and cluster-resolved pseudotime score distributions. Cluster ordered pseudotime identifies cyclical state progression in LCLs. **(E)** Pseudotime-ordered, phenotype-resolved expression of detected EBV latency genes and EBER transcripts in LCLs. **(F)** Integration of time-resolved FACS findings, scRNA-seq, and pseudotime dynamics support a model of conserved perpetual germinal center (GC) dynamics across EBV-immortalized cells *in vitro*.

Based on the observed dynamic heterogeneity, we re-analyzed scRNA-seq data from 3 LCLs representing infection with two EBV strains (B95-8 and M81). We first identified gene expression within cell subsets corresponding to the ICAM-1^Hi^/CD27^Lo^ and ICAM-1^Lo^/CD27^Hi^ FACS phenotypes across sample-integrated datasets (**Figures 3A, B**). Based on initial clustering, 18.7% of all sequenced cells corresponded to activated LZ-like B cells (ICAM-1^Hi^/CD27^Lo^) in LCLs. 18.0% of cells across LCLs matched the differentiated MBC phenotype (ICAM-1^Lo^/CD27^Hi^). These frequencies were approximately similar to those observed in the FACS experiments described previously, with higher frequencies of ICAM-1^Hi^/CD27^Lo^ cells observed *via* FACS relative to scRNA-seq. Further, differentially-expressed genes (DEGs) between activated and differentiated states were broadly consistent across samples. We further identified clusters that corresponded to transitional states between the activated and differentiated subsets. In contrast to our prior study of LCLs, we did not perform cell cycle marker gene regression during the data scaling step of the scRNA analysis, which preserved a distinct cluster of actively cycling cells. Cells in this cluster expressed numerous cell cycle genes and proliferation markers including *MKI67* and *CDK1* and were further resolved by mitotic phase (S, G_2_M; **Figure S2A**). These proliferating cells were consistent with the GC Dark Zone (DZ) state (42, 123). In addition to cycling cells, a cluster of a pre-GC activated B cell precursor to early memory B cells (AP-eMBCs) was also identified based on expression of *CCR6*, *CD22*, and other genes described in prior work (3, 15, 124) (**Figure 3C**, **S2B**). The four main phenotypes (MBC, AP-eMBC, DZ, and LZ) accounted for 86.4% of cells across LCL datasets.

Higher resolution clustering further refined cell states by immunoglobulin heavy chain class and cell cycle phase, for which we calculated pseudotime trajectories. Pseudotime graphs rooted within G_1_-phase differentiated memory cell clusters supported cyclical progression through the early stages of B cell activation, GC reaction (e.g., DZ←→LZ transitions), and eventual cell cycle exit and return to a G_1_-phase MBC state or (infrequently) pre-PB generation (**Figure 3D**). The apparent dynamic qualities of host gene expression were reflected in state-resolved EBV transcriptomes. On the basis of western blotting and qPCR, LCLs are conventionally described as having the latency III EBV program in which all EBNAs and LMPs are simultaneously expressed (24). However, pseudotime-ordered states portrayed a more nuanced picture of viral latency dynamics. As indicated by the fraction of cells expressing a given latency gene in each cluster, very few individual cells within LCLs simultaneously express all latency III genes. Actively dividing cells exhibited the broadest latency gene expression including elevated expression of EBNA1 (from the Q promoter), EBNA2 (from the C promoter), and EBNA-LP. With the notable exception of the small lytic cell population, the highest expression of LMP-1 was observed in the GC LZ-like NF-κB state. Along with EBER1 and EBER2, EBNA3A and EBNA3B expression from the C promoter exhibited transient upregulation in early cell cycle stages followed by decreases in G_2_M and G_0_/G_1_ states (**Figure 3E**). Collectively, the phenotype dynamics observed by FACS and conserved host and viral transcriptomic diversity led us to propose a model in which EBV infection perpetuates a core loop of host B cell entry, engagement, exit, and re-entry into a GC-like reaction *in vitro* (**Figure 3F**). In this model, persistent latent infection continuously drives the machinery of B cell adaptive immune responses in the absence of cognate antigen and restraint by other immune cell types.

**Figure 4:**
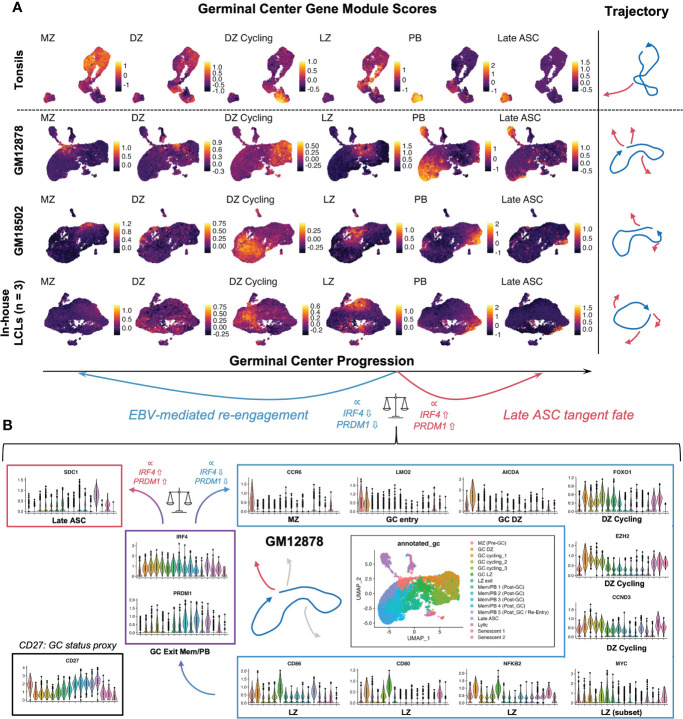
LCL subsets exhibit co-expression of genes associated with distinct GC phenotypes. **(A)** Gene module scores for mantle zone (MZ), dark zone (DZ), DZ cycling, light zone (LZ), plasmablast (PB), and antibody-secreting cell (ASC) states across tonsils and LCLs. Trajectories depict the path starting at the MZ phenotype and progressing through DZ, DZ cycling, LZ, post GC PB, and Late ASC. Blue trajectories depict the core GC and red trajectories represent exits from this dynamic. [MZ module = *CCR6, CD22, CD69, FCRL4, FCRL5, BANK1, MARCH1*; DZ module = *AICDA, FOXO1, CXCR4, AURKC, IL2RB*; DZ cycling module = *TCF3, EZH2, CCND3, E2F2, TP53, PLK4, BRCA1*; LZ module = *NFKB1, NFKB2, CD80, CD83, CD86, BCL2A1, EBI3, CD40, CR2, MIR155HG, ACKR3, MYO1C, MYC*; PB module *= PRDM1, XBP1, MZB1, TNFRSF17, CD27, CD38*; Late ASC = *SDC1*] **(B)** Detailed example of a GC-like cell paths in GM12878. Violin plots depicting key gene expression are presented to highlight the core GC dynamic (blue trajectory and box) as well as terminal differentiation to Late ASCs (red trajectory and box). The bifurcation point between ASC and GC re-entry trajectories is associated with cluster-resolved expression of *IRF4* and *PRDM1*, with lower expression of these genes associated with perpetual GC re-entry and higher expression associated with Late ASCs.

To further investigate the extent of EBV-induced GC characteristics in LCLs, we assayed the presence of cells with enriched signatures for distinct GC states based on literature-derived annotation (44, 125–135). By applying this analysis to scRNA-seq data from human tonsil samples as a reference for normal GCs, we confirmed that LCLs contained distinct subpopulations with characteristic of different pre-GC, GC, and post-GC cell states (**Figure 4A**). Likewise, we observed cells in LCLs with enriched signatures for classically described GC B cell genes and those used for molecular classification of GCB versus ABC DLBCL subtypes (125, 126). Surprisingly, LCL subsets with strong expression of pre-GC mantle zone (MZ) marker genes (*CCR6*, *CD22*, *CD69*, *FCRL4*, *FCRL5*, *BANK1*, *MARCH1*) had the highest correlation with the canonical GC B cell marker set (*BCL6, LMO2, MYBL1, MME, SERPINA9, GCSAM, DGKD, IL4R, SPI1, SH2B2, ALOX5, BCL7A, LCK, OGG1*). Moreover, LCLs further exhibited strong correlation of GCB and ABC DLBCL gene sets in contrast to tonsil tissue, which displayed anticorrelation of these sets as expected (**Figure S3A**). Collectively, these data suggest that the GC-like dynamics observed within LCLs are dysregulated with respect to normal GCs. In a detailed dissection of GM12878, we analyzed key gene expression within the EBV-driven perpetual GC. This analysis supported a potential path of cell state transitions from a mantle zone B cell phenotype through DZ entry, proliferation, LZ entry, LZ exit, and post-GC B cell differentiation, which indicated an apparent *IRF4*- and *PRDM1*-associated fate bifurcation between ASC development and GC re-entry *via* the MZ state (**Figure 4B**). In another example, we found that hallmark DZ genes (*FOXO1*, *CXCR4*) and LZ genes (*MYC*, *CD83*) were co-expressed in distinct subsets of GM18502 and that zone-mismatched genes had anticorrelated expression (**Figure S3B**). *LMO2* was generally co-expressed with other GC biomarkers (*MYBL1, SERPINA9, ALOX5, LRMP*) across tonsils and LCLs, although weaker correlations were observed in LCLs (**Figure S3C**). Interestingly, GC LZ genes (*BATF, CD40, CD83, CD86, CD274 (PD-L1), FAS*) were more strongly co-expressed with *ICAM1* in LCLs than in tonsils (**Figure S3D**), suggesting a bias toward LZ-like state enrichment in the context of EBV infection. Pseudotime analysis of classic GC B cell biomarker expression provided further support for EBV-driven GC-like dynamics in LCLs (**Figures S3E-L**), although we observed the striking absence of *BCL6* expression across LCLs (**Figure S3M**). As EBV has been shown to suppress *BCL6* expression and degrade BCL6 protein through EBNA3A, EBNA3C, and viral microRNAs (59, 136), the retention of certain germinal center features in LCLs implicates viral mechanisms that supplant BCL6 transcriptional regulation to some degree.

### Infrequent plasmablast formation, cellular quiescence, and viral reactivation define GC-tangent fates

**Figure 5:**
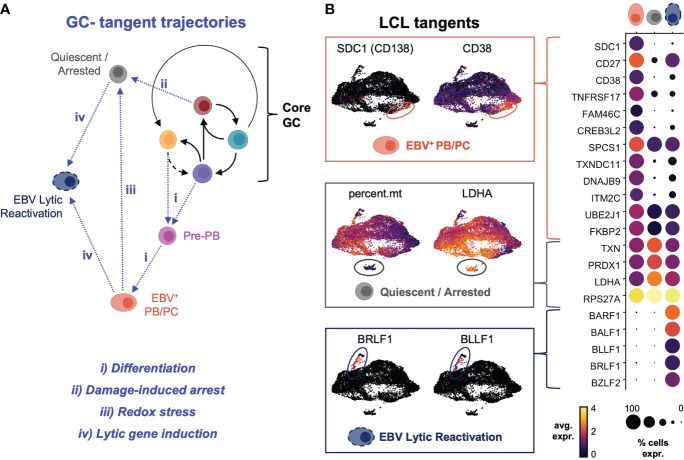
Plasma cell differentiation, growth arrest and quiescence, and viral reactivation define tangent fate trajectories arising from core GC dynamics. **(A)** Model of extra-GC tangent fate trajectories leading to additional phenotypes observed from LCL scRNA-seq data. Proposed triggers associated with each trajectory are annotated in blue. **(B)** Differentially expressed markers defining tangent phenotypes in LCLs (PB/PC = plasmablasts/plasma cells, quiescent/arrested cells, and lytic reactivation).

Sequencing data supported multiple possible fates for *CD27* ^+^ cells within LCLs (**Figure S4A**). Cells with an expression profile of *CCR6* ^+^/*CD27^+^/PRDM1^-^*/*SDC1^-^* were consistent with B cell re-entry into a GC-like reaction from the AP state. Other subsets expressing combinations of *CD27*, *CD38*, and *PRDM1* but not *CCR6* or *SDC1* were consistent with post-GC memory B cells and pre-plasmablasts. Notably, only a subset of post-GC *CD27* ^+^ B cells expressed *SDC1/CD138* and other late markers of dedication to the ASC fate. This ASC population and other subsets (typically with extremely high or low viral reads) appeared to exit conserved perpetual GC dynamics, which we characterized as “tangent” fate trajectories (**Figure 5A**). The terminally differentiated plasmablast tangent was further defined by elevated expression of *CD27*, *CD38*, *TNFRSF17/BCMA* and other genes previously identified from transcriptomic profiling of murine B cell subsets (137) (**Figure 5B**, top panel). These cells were distinguished by the highest expression of transcription factors (*PRDM1*, *XBP1*, *MZB1*) known to promote B cell differentiation to plasma cells (138, 139). Intriguingly, EBV^+^ plasma cells also expressed high levels of interferon response genes including, *IFI35*, *IFITM1*, *OAS1*, *MX1*, and *IFNG-AS1*, which enhances *IFNG* production in NK cells (140), and genes mediating redox stress (*TXNIP*, *TXNDC11*, *TXNDC15*), presumably in response to metabolic burdens associated with antibody secretion (141) (**Figure S4B**). Another tangent cluster of cells exhibited the fewest total and unique mRNAs but was enriched in transcripts for oxidative stress response genes (111) and ribosome subunit biogenesis (**Figure 5B**, middle panel). The low overall read counts suggested this cluster contained quiescent or growth-arrested cells, and functional enrichment of the top markers in these cells included ribosomal large subunit biogenesis (GO:0042273, FDR = 9.55e-11); regulation of G_2_/M phase transition (GO:1902749, FDR = 5.4e-5); hydrogen peroxide metabolism (GO:0042743, FDR = 0.0019); regulation of transcription from RNA Pol II promoter in response to hypoxia (GO:0061418, FDR = 0.0073); and negative regulation of nitrosative stress-induced intrinsic apoptotic signaling (GO:1905259, FDR = 0.039)Figure. A third distinct tangent fate had clear hallmarks of EBV lytic reactivation including expression of viral immediate (*BRLF1*), early (*BALF1, BARF1*), and late (*BZLF2, BLLF1*) lytic genes (**Figure 5B**, bottom panel). Consistent with prior reports, lytic cells within LCLs also displayed elevated expression *LMP1.* These reads may have derived from the truncated lytic LMP1 transcript (*lyLMP1*), which has been shown to be essential for successful virion release in reactivation (142, 143). These cells also expressed host genes (*NFATC1, MIER2*) known to mediate EBV reactivation (144) as well as several long non-coding RNAs (lncRNAs) and genes involved in chromatin remodeling and epigenetic silencing (*HOTAIRM1*, *REST*, *MALAT1*, *ZEB2-AS1*, *KCNQ1OT1*, *HOXB-AS3*, *DNMT3B*, *HDAC1*, *HDAC4*) (**Figure S5**).

### Atypical MBCs and autoimmune-like responsiveness to IL-21 and TLR7 agonism in EBV^+^ LCLs

**Figure 6:**
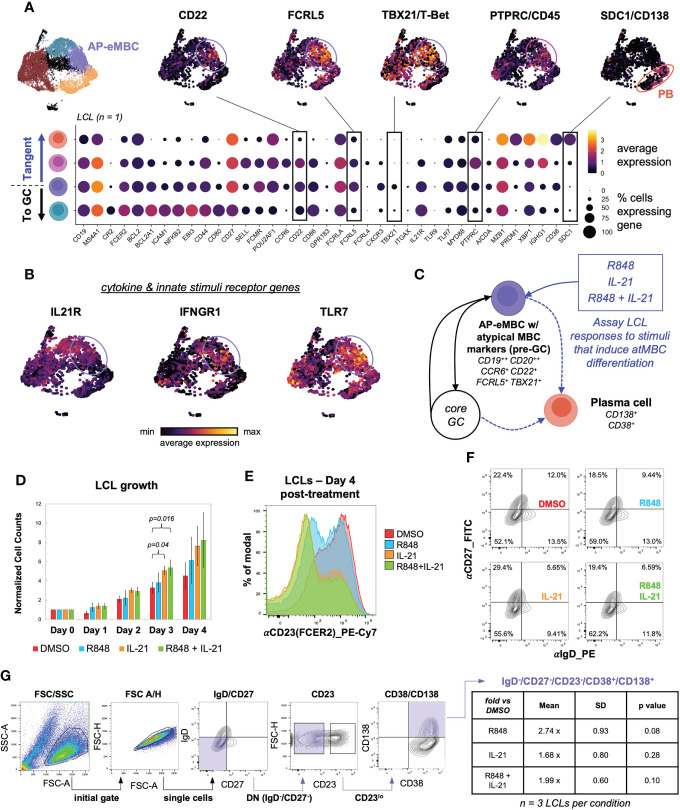
An EBV^+^ pre-GC activated state with hallmarks of T-bet^+^ memory B cells develops in a subset of LCLs. **(A)** AP-eMBC cluster cells within one in-house LCL dataset exhibits an atypical memory B cell (atMBC) phenotype prone to plasma differentiation. **(B)** Upregulation of genes encoding receptors for IL-21, IFNG, and TLR7 ligands within identified LCL atMBCs coincide with the AP-eMBC phenotype. **(C)** Model experimental design to stimulate plasma cell differentiation from AP-eMBC/atMBCs within LCLs. **(D)** Growth analysis of LCLs treated with 2 μg/mL TLR7 agonist R848 (blue), 10 ng/mL IL-21 (orange), or both (green) versus control treatment (0.1% DMSO, red). Data are presented as mean +/- standard deviation cell counts with intra-replicate normalization to Day 0 across 3 biological replicates per condition. Statistically significance of differences (Day 3 IL-21 vs. DMSO, p = 0.04 and Day 3 R848+IL-21 vs. DMSO, p = 0.016) were calculated using Welch’s t test (two-tailed, paired). **(E)** Representative CD23 (FCER2) expression in LCLs at Day 4 by treatment group. **(F)** CD27 and IgD staining of LCLs at Day 4 by treatment group to evaluate the frequency of EBV-infected double-negative (DN) B cells (IgD^-^/CD27^-^). **(G)** Gating strategy to identify DN B cell-derived plasma cells (IgD^-^/CD27^-^/CD23^-^/CD38^+^/CD138^++^) and quantification by treatment relative to control treated LCLs at Day 4. Statistical significance was evaluated by Welch’s two-tailed t-test.

We recently found that EBV infection induced non-canonical gene expression changes within T-bet^+^ atypical memory B cells (atMBCs) (15), which have been identified as expanded pathogenic cell subsets in diseases including multiple sclerosis (MS) (10), systemic lupus erythematosus (SLE) (12, 145), and rheumatoid arthritis (RA) (146). This atMBC response to infection included upregulation of pro-inflammatory signature and aberrant expression of neuronal lineage genes. Based on these findings from *de novo* infection, we questioned whether cells consistent with EBV^+^ atMBCs were present in LCLs. One of the three scRNA-seq datasets from LCLs generated in our lab contained a clear population of atMBCs based on expression of *TBX21* (T-bet) and *CXCR3* in addition to genes encoding BCR regulating receptors, including *FCRL4* and *FCRL5* (**Figures 6A**, **S6A**) (147, 148). Notably, these EBV^+^ atMBCs were highly correlated with genes defining the pre-GC activated B cell precursor to MBC (AP-eMBC) state (*CCR6, POU2AF1, CD22*). EBV^+^ atMBCs were further distinguished by elevated expression of genes encoding receptors for key cytokines (*IL21R*, *IFNGR1*) and innate stimuli (*TLR7* and its downstream signaling adapter gene *MYD88*) known to mediate atMBC differentiation, including pathogenic responses in numerous human autoimmune diseases (**Figure 6B**) (100, 104, 149). Re-analysis of publicly available data from two additional LCLs (GM12878 and GM18502) likewise revealed conserved co-expression of *IL21R*, *IFNGR1*, *TLR7*, *CCR6*, *CD22*, *FCRL5*, *TBX21*, *CXCR3*, and *CD11c/ITGAX*, indicating the presence of EBV^+^ atMBC/AP-eMBC populations in three of five analyzed LCLs (**Figure S6B**).

TLR7 agonism and IL-21 treatment have been shown to elicit expansion and differentiation of human T-bet^+^ atMBCs *in vitro* and analogous murine age-associated B cells (ABCs) *in vivo* (100, 149). Given these findings, we hypothesized that EBV^+^ atMBCs might exhibit similar responses upon stimulation (**Figure 6C**). To test this, we treated LCLs with the TLR7 agonist R848 (resiquimod, 2 μg/mL), IL-21 (10 ng/mL), or both and measured cell growth and surface marker expression relative to a control treatment (0.2% DMSO). Despite having a high basal proliferation rate (~24h doubling time), LCLs stimulated with IL-21 (with or without R848) exhibited accelerated proliferation and higher cell densities than controls within three days of treatment (n = 3, IL-21 vs DMSO p = 0.04; R848+IL-21 vs. DMSO p = 0.016, two-tailed paired Welch’s t-test) (**Figure 6D**). The most prominent response we observed in stimulated LCLs was a marked reduction in CD23 (FCER2) expression, with the greatest decrease observed in IL-21 and R848+IL-21-treated LCLs (IL-21 vs. DMSO p = 0.00021; IL-21+R848 vs. DMSO p = 3.51x10^-5^, two-tailed Welch’s t-test of CD23 distribution geometric means, n = 4) (**Figure 6E**, **S7A**). IL-21 and R848 stimulation also led to modest increases in the percentage of IgD^-^/CD27^-^ (double-negative, DN) B cells relative to the control treatment (**Figure 6F**). Based on prior studies on stimulated atMBC differentiation and the pathogenicity of atMBC-derived plasmablasts (PBs) (12, 101, 150), we assayed the frequency of DN-derived PBs (defined as IgD^-^/CD27^-^/CD23^-^/CD38^+^/CD138(SDC1)^+^ cells) across LCL treatment groups. Using a sequential gating strategy, we observed higher frequencies of autoimmune-associated DN-derived PBs in LCLs treated with R848, IL-21, or R848+IL-21 relative to control LCLs, although these differences were not statistically significant. R848-treated LCLs (n = 3, 2.74 ± 0.93 fold more DN PBs vs. DMSO, two-tailed Welch’s t-test p = 0.08) had the highest frequency of DN-derived PBs (**Figure 6G**). Intriguingly, even unstimulated LCLs exhibited a significantly higher DN B cell frequency than uninfected peripheral B cells (74.9 ± 2.3% vs 17.8 ± 1.3%, Welch’s t-test p = 0.008, n = 2), though these data do not represent donor-matched measurements (**Figure S7B**).

### Proliferative CXCR3^+^ B cells are induced during *de novo* EBV infection

**Figure 7:**
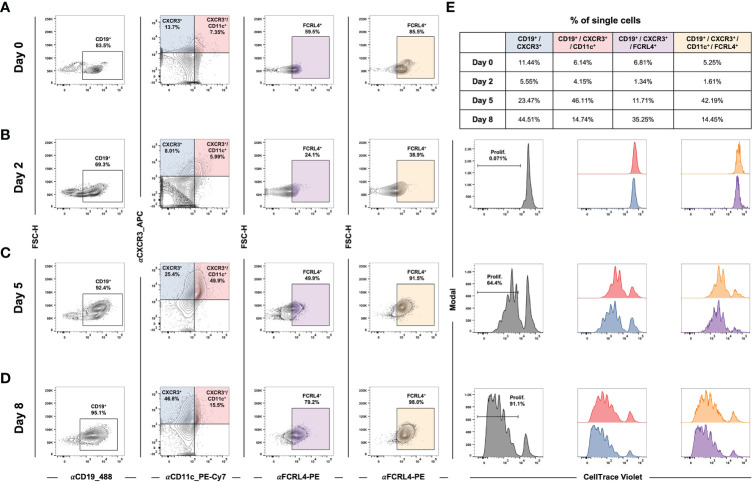
EBV *de novo* infection of peripheral B cells induces a CXCR3^+^/CD11c^+^/FCRL4^+^ population that exhibits classic hyperproliferation. **(A)** Gating for CD19^+^/CXCR3^+^/CD11c^+^/FCRL4^+^ cells within uninfected B cells enriched from PBMCs. **(B)** Gating as in A) at Day 2 post-EBV infection. **(C)** Gating for Day 5 post-EBV infection. **(D)** Gating for Day 8 post-EBV infection. **(E)** Cell proliferation and distribution of division number over time by gated populations (all CD19^+^, CD19^+^/CXCR3^+^/CD11c^+^, and CD19^+^/CXCR3^+^/CD11c^+^/FCRL4^+^).

In addition to studying atMBC responses to stimuli in the context of established EBV latency, we asked whether *de novo* infection itself was sufficient to induce an atMBC-like phenotype. Specifically, recent work has reported an association between EBV infection and a neuroinvasive, pathogenic subset of T-bet^+^/CXCR3^+^ B cells identified within clinically isolated syndrome (CIS)/multiple sclerosis (MS) patients (151–153). To address this possibility, we performed time-resolved FACS experiments to determine whether CXCR3-expressing B cells were induced during the early stages of EBV infection. Infection of enriched peripheral blood B cells with the B95-8 EBV strain led to the development of a CD19^+^/CXCR3^+^/CD11c^+^ cell population within five days *in vitro* (**Figures 7A-D**, **Figure S7C**). After the first several days of infection, the percentage of CD19^+^/CXCR3^+^/CD11c^+^ that also expressed FCRL4 (an inhibitory receptor and EBV-induced host biomarker (122)) progressively increased (**Figures 7A-D**, third column). In single cells gated from two biological donors, the average frequency of CD19^+^/CXCR3^+^/CD11c^-/+^/FCRL4^+^ gated cells was 38.6 ± 15.7% at day 8 post-infection compared to 9.8 ± 3.2% of cells prior to infection (n = 2 LCLs, two-tailed Welch’s t-test p = 0.224). Notably, CD19^+^/CXCR3^+^/CD11c^+/-^/FCRL4^+^ cells underwent EBV-induced hyperproliferation and exhibited similar cell division profiles relative to the total CD19^+^ population based on cell tracking dye dilution over time (**Figure 7E**, **S7C**).

## Discussion

Single-cell technologies provide powerful means to examine how EBV manipulates host cell programming to achieve viral replication. In the present work, we have used these methods to identify and study cellular diversity that arises *via* EBV infection. Our findings yield new insight into dynamic virus-driven cell heterogeneity within lymphoma models and provide a framework to explore the functional significance of EBV infection within T-bet^+^ atypical B cells that may promote pathogenic features of this cellular niche in autoimmunity and chronic infection.

**Figure 8:**
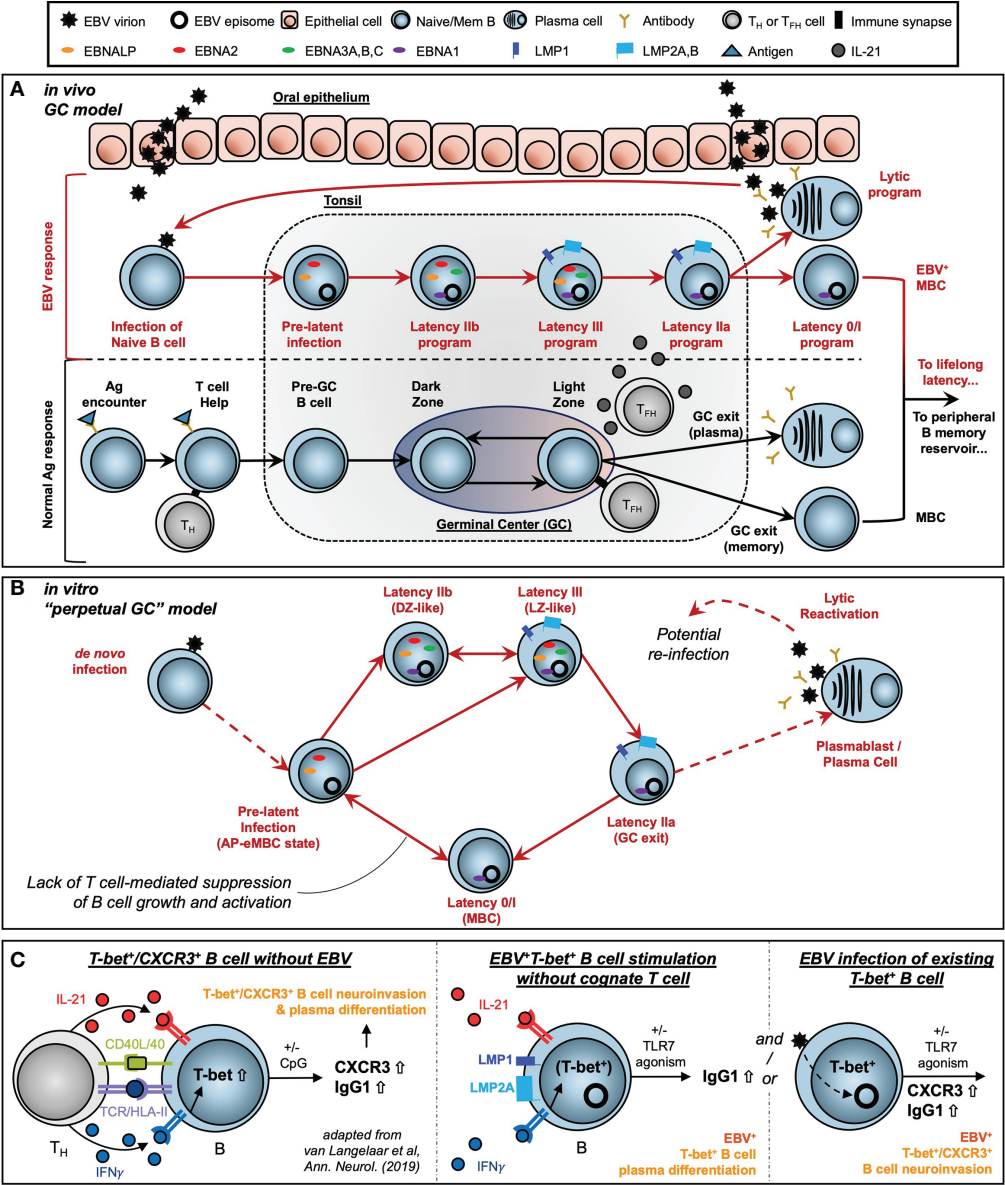
Models of EBV-driven germinal center dynamics and possible roles in priming of T-bet^+^ atypical MBC pathogenesis. **(A)** The Germinal Center (GC) model of EBV infection *in vivo*. **(B)** A model of perpetual GC dynamics within EBV-immortalized B cells *in vitro*. **(C)** Models of atMBC behaviors associated with pathogenic autoimmunity and potential EBV-induced priming of these responses.

Extensive work by Thorley-Lawson and colleagues has supported the development of the Germinal Center (GC) model of EBV infection, which accounts for an *in vivo* route to EBV latency establishment within the peripheral MBC reservoir (41, 42, 56, 154). Subsequent studies by our lab and others (24, 32, 43, 122, 155–157) have provided refinements to the GC model including the correspondence of distinct viral latency gene expression programs with the stages of B cell response to antigen (**Figure 8A**). These include a pre-latent phase (mimicking pre-GC B cells); Latency IIb, during which all six EBNAs are expressed and cells rapidly proliferate (GC DZ-like); Latency III, in which the LMPs are additionally expressed and promote cell survival (GC LZ-like); and Latency IIa, in which EBNA expression is downregulated concomitant with GC exit. At a high level, the GC model underscores the capacity of EBV to induce programmed B cell responses in the absence of BCR-cognate antigen and direct T_H_ cell engagement. However, the potent immunogenicity of the latency gene products and prevalence of EBV-specific T cells provides a selective pressure for infected MBCs to adopt restricted latency *in vivo*, in which only the episome-maintaining EBNA1 and non-coding EBERs and miRNAs (Latency I) or EBERs and miRNAs only (Latency 0) are expressed.

By contrast, T cell-mediated suppression of EBV^+^ MBCs may be attenuated in patients with compromised or suppressed immune systems. Unchecked proliferation of EBV^+^ MBCs in these contexts can lead to the development of virus-associated Diffuse Large B Cell Lymphomas (DLBCLs) including post-transplant lymphoproliferative disease (PTLD) in organ transplant recipients (75). In this regard, EBV-immortalized *in vitro* systems provide useful models to study virus-driven lymphomagenesis associated with these diseases. We found that distinct subsets of B cells within LCLs undergo dynamic interconversion to sustain equilibrium distributions of activated and differentiated states. Critically, memory cells (ICAM-1^Lo^/CD27^Hi^) did not constitute a dead end for replication but could instead develop into the activated phenotype (ICAM-1^Hi^/CD27^Lo^) with greater proliferative potential. We further utilized pseudotime analysis of scRNA-seq to demonstrate that these *in vitro* dynamics are part of a perpetual cycle of B cell GC reaction engagement (**Figure 8B**). This perpetual GC was conserved across normal B cells infected with different strains of EBV (B95-8, M81). Another recent study (158) developed additional scRNA-seq data from LCLs generated with Type 1 Mutu and Type 2 BL5 EBV strains. We did not conduct a formal analysis of these datasets, however the conservation of GC-like states in cells immortalized by these strains is clear [MBCs = clusters 4, 5; AP-eMBC = cluster 2; Act. LZ = cluster 1; cycling DZ = clusters 0, 3, and 7; plasmablasts = cluster 6; lytic and pre-lytic cells = clusters 8, 9, and 10 – refer to Figure 11 in Bristol et al. (158)].

Our data demonstrate that EBV mediates B cell activation and subsequent differentiation *via* GC-like dynamics. Virus-induced simulation of GC transcriptional programs and biomarkers is especially noteworthy since EBV strongly suppresses the expression and functions of the master transcriptional regulator of the germinal center, BCL6 (59, 136, 159). The ability of EBV infection to phenocopy elements of the GC reaction in the absence of BCL6 may be promoted at least partially by virus-induced epigenetic alterations that parallel centrocyte (LZ B cell) *cis* and *trans* regulatory control at the *BCL2A1* locus, which we have recently reported (160). While *in vitro* EBV-induced LCL formation is well understood in the context of the functions of viral oncoproteins, it is intriguing to consider the contributions of B cell-intrinsic biology to immortalization in culture. Such aspects include the continuous engagement of GC B cell transcriptional programs and, more generally, the retained cellular plasticity that underlies adaptive immune responses and memory. While important facets of GC B cells and their functions are evident within LCLs, our data indicate that there are discrepancies not only between LCLs and tonsillar GCs but also with respect to classically defined DLBCL subtypes. Specifically, cells within LCLs – at least those derived from unsorted peripheral B cells – can display co-expression of genes characteristic of both GCB-DLBCLs and ABC-DLBCLs. In this context, future work is needed to assess whether B cells at different developmental or functional stages that are transformed by EBV might retain cell of origin characteristics. Whether the heterogeneity and state transitions of the *in vitro* perpetual GC are defining features of EBV^+^ lymphomas *in vivo* is currently unclear, however it has been found that GC dynamics are de-synchronized in Follicular Lymphoma (FL) (7), which is rarely EBV^+^. Thus, future studies that examine clinical samples will be essential to determine whether B cells with unregulated GC dynamics or even spatial organization are present across a spectrum of EBV^+^ lymphoid malignancies.

The exquisite adaptation of EBV to modulate host cell programs highlights the importance of using single-cell approaches to resolve consequences of viral infection within particular B cell subsets. This is especially relevant for T-bet^+^ atMBC pools, which are enriched with autoreactive clones (161, 162). Pathogenic atMBC expansion (and differentiation to plasma cells) elicited by cytokines and innate stimuli constitutes a common feature of chronic infection with HIV (106) and *Plasmodium falciparum (* 105) and an array of diseases of autoimmunity or immune dysregulation including multiple sclerosis (MS) (10), systemic lupus erythematosus (SLE) (12), rheumatoid arthritis (RA) (146), and primary Sjögren’s Syndrome (pSS) (163). Notably, associations with EBV have been identified in many of the same diseases in which atMBCs are dysfunctional. Thus, our scRNA-seq evidence for atMBCs within LCLs and corresponding tangent fate trajectories including plasmablast formation prompted us to use LCLs as a model system to study possible roles of latent EBV infection in aspects of B cell-mediated autoimmunity. We also examined the potential role that *de novo* EBV infection may play in promoting the development of CXCR3^+^ atMBCs identified in patients with MS. This interest was rooted in several distinct lines of evidence. First, we recently reported that *de novo* EBV infection within atMBCs (*TBX21*/T-bet^+^, *ITGAX*/CD11c^+^, *FCRL4* ^+^, *FCRL5* ^+^) induced elevated expression of inflammatory mediators and – unexpectedly – neuronal lineage genes involved in nervous system development and axon guidance (15). Second, there is strong etiological support for the importance of EBV seropositivity during late adolescence or early adulthood – but not childhood – in the development of MS (94–96). Paired with studies demonstrating the accumulation of T-bet^+^ atMBCs with age (100), we see a convergence of epidemiological and functional studies that implicates a pathogenic role for *de novo* EBV infection of atMBCs. Finally, recent reports by van Langelaar and colleagues have described an enriched subset of CXCR3^+^ neuroinvasive B cells associated with elevated EBV viral loads in MS patients (151, 152). Based on their findings, van Langelaar et al. developed a model in which MS patient B cell engagement with a cognate T cell (CD40/CD40L and TCR/Ag-MHC class II recognition) in conjunction with IL-21 and especially IFNγ stimulation induces T-bet expression and subsequent CXCR3 upregulation (**Figure 8C**, left panel). In this model, CXCR3 promotes T-bet^+^ B cell migration from secondary lymphoid tissue to the brain *via* the periphery, consistent with this chemokine receptor’s essential role in T cell (164) and ASC (165) neuromigration. Additional innate stimulation *via* TLR agonism then mediates the conversion of T-bet^+^ B cells to antibody-secreting cells.

In the latent infection context, EBV^+^ atMBCs may develop from EBV^+^ MBCs that are subsequently stimulated to express T-bet by IFNγ secreted by proximal T cells, possibly in response to unrelated infections. These EBV^+^ atMBCs may then be primed for differentiation in response to IL-21 and/or TLR7 agonism without cognate engagement with T cells (**Figure 8C**, middle panel). This scenario is conceivable for infected B cells that express both LMP1, which mediates CD40 signaling, and LMP2A, which downregulates MHC class II expression and mimics a constitutively activated BCR. The observed significant enrichment of an autoimmune-associated DN B cell phenotype (CD19^+^/IgD^-^/CD27^-^) in LCLs versus uninfected peripheral B cells provides *in vitro* evidence that infected atMBCs may develop in the course of EBV latency establishment. We also assayed the effects of TLR7 agonism and IL-21 stimulation of LCLs on cell proliferation and the induction of plasma cells, both of which are characteristics of atMBCs in autoimmunity. Treatment of LCLs with IL-21 (with or without R848) led to marked reduction of CD23 expression. This response is particularly notable, given that CD23^-^ GC B cells from human tonsils have been identified as precursors to plasma cells (166) and that expansion and differentiation of CD23^-^ DN B cells has been observed in SLE (145, 167, 168). Treatment with IL-21 or R848 + IL-21 also yielded modest but significant increases in LCL growth, consistent with responses of atMBC niches including DN B cells to these stimuli (149). In this regard, it is noteworthy that *TBX21(T-bet)* ^+^ atMBCs in LCL scRNA-seq data consistently exhibited elevated expression of *IL21R* and *TLR7*, inhibitory BCR co-receptors, and pre-GC AP-eMBC signatures. These facets are consistent with defined characteristics of atMBCs common to chronic infections and autoimmunity including BCR hyposensitivity (anergy) and extrafollicular activation by innate stimuli (11, 169). Whether antigen-independent B cell activation enacted through EBV infection predisposes poor affinity maturation – another hallmark of autoreactive atMBCs – remains to be studied. In contrast to the robustness of CD23 downregulation upon IL-21 treatment of LCLs, we observed only subtle increases (not statistically significant) in the frequency of EBV^+^ IgD^-^/CD27^-^/CD23^-^/CD38^+^/CD138^+^ cells relative to controls. We speculate that the effect of these stimulations on cell growth may be muted in LCL models due to their high basal rates of proliferation. Moreover, plasma cell formation within LCL populations is generally uncommon, since viral EBNA3A and EBNA3C proteins suppress plasma cell differentiation within roughly two weeks of latency establishment *via* epigenetic modifications of the *PRDM1* (Blimp-1) and *CDKN2C* (p18^INK4C^) loci (59). These viral countermeasures to circumvent terminal differentiation likely contribute to the minimal increase in DN-derived PBs upon stimulation despite the significant downregulation of CD23. Thus, we emphasize that LCLs without additional experimental modifications may have limited utility for studies of EBV^+^ MBC responses to stimuli in the context of autoimmunity. The fact that plasmablasts arise at all within LCLs raises questions as to what mechanisms might enable EBV^+^ B cells to overcome EBNA3-mediated epigenetic suppression of plasma cell formation – and whether they occur within EBV^+^ atMBCs *in vivo*.

In the *de novo* infection context, we found that EBV increases CXCR3 (as well as CD11c and FCRL4) expression on peripheral B cells. Moreover, EBV^+^ CD19^+^/CXCR3^+^/CD11c^+^/FCRL4^+^ cells and parental CD19^+^ populations proliferated to a similar extent in response to infection. Consistent with these findings, EBV infection of existing T-bet^+^ atMBCs may provide a stimulus that facilitates the reported expansion and neuroinvasive phenotype of CXCR3^+^ atMBCs (**Figure 8C**, right panel). As EBV virion entry is known to upregulate TLR7 expression and pathway sensitivity (170), it is possible that newly infected atMBCs may be primed for differentiation to plasmablasts triggered by recognition of exogenous nucleic acids from EBV itself or other infectious agents. In this case, it is alluring to speculate that differentiation of recently infected atMBCs upon innate stimulation may rapidly promote the initiation of viral reactivation through XBP1 and PRDM1 transactivation of the master EBV lytic regulator *BZLF1*.

Echoing the distinctions between the GC model of infection and conventional B cell specific immunity, EBV may prime atMBCs for pathogenic responses in *de novo* infection and/or atMBCs derived from latently infected MBCs without explicit requirements for antigen-specific activation or direct T cell engagement. While the data and models presented here provide a starting point to dissect viral involvement in aspects of atMBC-mediated autoimmunity, this is currently a field with many more open questions than definitive answers. Future functional and mechanistic studies are clearly required to address these questions and test the proposed models. We expect the presented single-cell analysis and experiments as well as future high-resolution studies will be essential to dissect EBV-mediated dysregulation of B cell compartments in lymphoproliferative malignancies, chronic infections, and autoimmune diseases.

## Data availability statement

The datasets presented in this study can be found in online repositories. The names of the repository/repositories and accession number(s) can be found below: https://www.ncbi.nlm.nih.gov/geo/, GSE158275; https://www.ncbi.nlm.nih.gov/geo/, GSE126321; https://www.ncbi.nlm.nih.gov/geo/, GSE159674

## Ethics statement

The primary B cells and LCLs used in this study were prepared from buffy coats obtained through the Gulf Coast Regional Blood Center from anonymous donors. These biological samples lacked all HIPAA identifiers and PHI and the studies were thus considered as non-human subjects research and approved by a Duke University IRB (eIRB #Pro00006262).

## Author contributions

ES and NR-V share first authorship of this work. ES, NR-V, GH, and ML contributed to the conception and design of the study. ES, NR-V, and GH performed the experiments and acquired the data. ES, NR-V, GH, and ML analyzed the data. ES and NR-V wrote the initial draft of the manuscript. GH wrote sections of the manuscript. All authors contributed to the article and approved the submitted version.

## Funding

This study was supported by NIH funding from the National Institute of Dental and Craniofacial Research (NIDCR, #R01DE025994). ES wishes to acknowledge support from the Duke University Viral Oncology Training Grant (NIH, #T32CA009111) and an American Cancer Society postdoctoral fellowship (ACS, PF-21-084-01-DMC).

## Acknowledgments

We would like to thank the Luftig Lab members – especially Katherine Willard – for thoughtful feedback during the development of this study and in drafting the manuscript and figures. We also wish to thank the anonymous blood donors whose samples made this work possible.

## Conflict of interest

The authors declare that the research was conducted in the absence of any commercial or financial relationships that could be construed as a potential conflict of interest.

## Publisher’s note

All claims expressed in this article are solely those of the authors and do not necessarily represent those of their affiliated organizations, or those of the publisher, the editors and the reviewers. Any product that may be evaluated in this article, or claim that may be made by its manufacturer, is not guaranteed or endorsed by the publisher.

## Supplementary material

Supplementary Figure 1 Additional LCL replicates for FACS populations and sorting. (A) Biological replicates for ICAM-1/CD27 FACS staining. (B) Phenotype composition in LCLs is dependent on cell density. (C) Biological replicates for time-resolved assays of ICAM-1 and CD27 in sorted LCL fractions. Top row in each replicate: ICAM-1^Hi^/CD27^Lo^. Middle row in each replicate: ICAM-1^Lo^/CD27^Lo^. Bottom row in each replicate: ICAM-1^Lo^/CD27^Hi^. The box inset depicts a technical artifact arising from FACS buffer contamination that affected the observed CD27/ICAM1 distribution from days 3-5. (D) Replicate experiments performed with increased gating stringency to exclude the possibility of phenotype contamination leading to the observed recovery of parental line distributions.

Supplementary Figure 2 Additional marker genes in DZ and AP-eMBC states. (A) Key marker genes in cycling (DZ-like) cells across LCLs. Data are presented for three LCLs generated in-house (left column) and commercially available LCLs (GM12878, middle; GM18502, right) originally reported by Osorio et al (112). (B) Key marker genes in AP-eMBC cells across LCLs. Data are presented for three LCLs generated in-house (left column) and commercially available LCLs (GM12878, middle column; GM18502, right column) originally reported by Osorio et al (112).

Supplementary Figure 3 GC B cell and DLBCL gene expression in LCLs and tonsils. (A) GC B cell biomarker scoring in tonsil and LCL scRNA-seq datasets (left column). Gene module correlations between Classic GC, DZ, DZ cycling, and LZ markers (middle column). Correlation between GCB-DLBCL and ABC-DLBCL module scores. [Classic GC module = *BCL6, LMO2, MYBL1, MME, SERPINA9, GCSAM, DGKD, IL4R, SPI1, SH2B2, ALOX5, BCL7A, LCK, OGG1*; DZ module = *AICDA, FOXO1, CXCR4, AURKC, IL2RB*; DZ cycling module = *TCF3, EZH2, CCND3, E2F2, TP53, PLK4, BRCA1*; LZ module = *NFKB1, NFKB2, CD80, CD83, CD86, BCL2A1, EBI3, CD40, CR2, MIR155HG, ACKR3, MYO1C, MYC*] (B) Co-expression of canonical GC DZ markers (*FOXO1*, *CXCR4*) and GC LZ markers (*MYC*, *CD83*) in cell subsets within GM18502. (C) Co-expression of *LMO2* and other classic GC B cell markers (*MYBL1, SERPINA9, ALOX5, LRMP*) across tonsil and LCL scRNA-seq datasets. (D) Co-expression of *ICAM1* with LZ GC B cell markers (*BATF, CD40, CD83, CD86, CD274, FAS*) across tonsil and LCL scRNA-seq datasets. (E) Anti-correlated expression of the GC LZ marker *CD83* and GC DZ marker *CXCR4* across 3 in-house LCL scRNA-seq datasets. (F) Pseudotime-resolved anticorrelated expression of DZ (*AICDA*, *CXCR4*) versus LZ (*CD83*, *MIR155HG*) genes. Cells are ordered in pseudotime and colored by high-resolution clusters as shown in -E. (G) Cluster-resolved average expression of genes upregulated in GC B cells relative to activated blood B cells (curated from Alizadeh et al, 2000). (H) Pseudotime- and cluster-resolved expression of MAP4K2 (Germinal Center Kinase, identified from Alizadeh et al, 2000). (I) Expression of GC DZ-associated transcriptional regulators (*TCF3*, *EZH2*) and their transcriptional targets upregulating cell proliferation (*CCND3*, *E2F2*) in pseudotime. (J) *MYC* and *NFKB* family transcription factor expression in pseudotime peaks in GC LZ-like cells. (K) Genes with known roles in constraining the GC reaction exhibit elevated expression in DZ and DZ cycling cells. (L) Expression of GC B cell biomarker genes *BCL7A, MYBL1, and LMO2* across in-house LCLs. (M) Expression of the GC B cell biomarker *MME* and the transcriptional regulators *MEF2B* and *BCL6* is limited or absent from EBV-induced GC-like properties in LCLs.

Supplementary Figure 4 Distinct *CD27^+^* subsets and key marker genes in plasma cells across LCLs. (A) UMAP and upset plots of *CD27* co-expression with markers of pre-GC early activation (*CCR6*), post-GC memory B cells/pre-plasmablasts (*CD38, PRDM1*), and late ASCs (*SDC1*) across LCLs. Upset plots depict cells with co-expression at levels greater than or equal to the 25^th^ percentile for each gene’s respective distribution across the given dataset. (B) Top markers within late plasma cells (ASCs). Data are presented for three LCLs generated in-house (left column) and commercially available LCLs (GM12878, middle column; GM18502, right column) originally reported by Osorio et al (112).

Supplementary Figure 5 Key marker genes in lytic cells across LCLs. Data from differentially expressed genes of interest are presented for three LCLs generated in-house.

Supplementary Figure 6 Expression signature of atMBCs within LCLs. (A) Pearson R correlations for single-cell co-expression of the atMBC markers *TBX21*, *FCRL5*, and *CXCR3* in LCLs. (B) Data from differentially expressed genes of interest are presented for three LCLs generated in-house and commercially available LCLs (GM12878, GM18502) originally reported by Osorio et al (112). Three of five LCL datasets contain cells consistent with atMBCs exhibiting elevated expression of key receptors that mediate atMBC responses to stimuli in addition to AP-eMBC characteristics (identified by green circles). Cells with AP-eMBC characteristics but lacking definitive atMBC gene expression in the remaining two LCLs are also highlighted (blue circles).

Supplementary Figure 7 Additional replicates for FACS studies of EBV-associated autoimmune phenotypes in LCLs and *de novo* infection. (A) FACS replicates for CD23 (FCER2) downregulation upon stimulation with R848 and/or IL-21. (B) FACS staining of IgD and CD27 in uninfected peripheral B cells to identify putative double negative (DN) B cell frequencies. Samples were prepared *via* B cell enrichment (negative isolation) from PBCMs. Data are presented for two donors. (C) Biological replicate for cell proliferation and CXCR3, CD11c, FCRL4 staining in *de novo* infection experiments.

## References

[1] King HW Orban N Riches JC Clear AJ Warnes G Teichmann SA. Single-cell analysis of human B cell maturation predicts how antibody class switching shapes selection dynamics. Sci Immunol (2021) 6(56):eabe6291. doi: 10.1126/sciimmunol.abe6291 33579751

[2] Ranzoni AM Tangherloni A Berest I Riva SG Myers B Strzelecka PM. Integrative single-cell RNA-Seq and ATAC-Seq analysis of human developmental hematopoiesis. Cell Stem Cell (2021) 28(3):472–87.e7. doi: 10.1016/j.stem.2020.11.015 33352111 PMC7939551

[3] Glaros V Rauschmeier R Artemov AV Reinhardt A Ols S Emmanouilidi A. Limited access to antigen drives generation of early B cell memory while restraining the plasmablast response. Immunity (2021) 54(9):2005–23.e10. doi: 10.1016/j.immuni.2021.08.017 34525339 PMC7612941

[4] Stewart A Ng JC-F Wallis G Tsioligka V Fraternali F Dunn-Walters DK. Single-cell transcriptomic analyses define distinct peripheral B cell subsets and discrete development pathways. Front Immunol (2021) 12:602539. doi: 10.3389/fimmu.2021.602539 33815362 PMC8012727

[5] Madissoon E Wilbrey-Clark A Miragaia R Saeb-Parsy K Mahbubani K Georgakopoulos N. scRNA-seq assessment of the human lung, spleen, and esophagus tissue stability after cold preservation. Genome Biol (2020) 21(1):1–16. doi: 10.1186/s13059-019-1906-x PMC6937944 31892341

[6] Steen CB Luca BA Esfahani MS Azizi A Sworder BJ Nabet BY. The landscape of tumor cell states and ecosystems in diffuse large B cell lymphoma. Cancer Cell (2021) 39(10):1422–37. doi: 10.1016/j.ccell.2021.08.011 PMC9205168 34597589

[7] Milpied P Cervera-Marzal I Mollichella M-L Tesson B Brisou G Traverse-Glehen A. Human germinal center transcriptional programs are de-synchronized in B cell lymphoma. Nat Immunol (2018) 19(9):1013–24. doi: 10.1038/s41590-0181-4 30104629

[8] Andor N Simonds EF Czerwinski DK Chen J Grimes SM Wood-Bouwens C. Single-cell RNA-Seq of follicular lymphoma reveals malignant B-cell types and coexpression of T-cell immune checkpoints. Blood J Am Soc Hematol (2019) 133(10):1119–29. doi: 10.1182/blood-2018-08-862292 PMC6405336 30591526

[9] Holmes AB Corinaldesi C Shen Q Kumar R Compagno N Wang Z. Single-cell analysis of germinal-center B cells informs on lymphoma cell of origin and outcome. J Exp Med (2020) 217(10):e20200483. doi: 10.1084/jem.20200483 32603407 PMC7537389

[10] Ramesh A Schubert RD Greenfield AL Dandekar R Loudermilk R Sabatino JJ. A pathogenic and clonally expanded B cell transcriptome in active multiple sclerosis. Proc Natl Acad Sci (2020) 117(37):22932–43. doi: 10.1073/pnas.2008523117 PMC7502747 32859762

[11] Holla P Dizon B Ambegaonkar AA Rogel N Goldschmidt E Boddapati AK. Shared transcriptional profiles of atypical B cells suggest common drivers of expansion and function in malaria, HIV, and autoimmunity. Sci Adv (2021). doi: 10.1126/sciadv.abg8384 PMC8153733 34039612

[12] Wang S Wang J Kumar V Karnell JL Naiman B Gross PS. IL-21 drives expansion and plasma cell differentiation of autoreactive CD11chiT-bet+ B cells in SLE. Nat Commun (2018) 9(1):1–14. doi: 10.1038/s41467-018-03750-7 29717110 PMC5931508

[13] Mathew NR Jayanthan JK Smirnov IV Robinson JL Axelsson H Nakka SS. Single-cell BCR and transcriptome analysis after influenza infection reveals spatiotemporal dynamics of antigen-specific B cells. Cell Rep (2021) 35(12):109286. doi: 10.1016/j.celrep.2021.109286 34161770 PMC7612943

[14] Scharer CD Patterson DG Mi T Price MJ Hicks SL Boss JM. Antibody-secreting cell destiny emerges during the initial stages of B-cell activation. Nat Commun (2020) 11(1):1–14. doi: 10.1038/s41467-020-17798-x 32778653 PMC7417592

[15] SoRelle ED Dai J Reinoso-Vizcaino NM Barry AP Chan C Luftig MA. Time-resolved transcriptomes reveal diverse B cell fate trajectories in the early response to Epstein-Barr virus infection. Cell Rep (2022) 40(9):111286. doi: 10.1016/j.celrep.2022.111286 36044865 PMC9879279

[16] Yewdell WT Smolkin RM Belcheva KT Mendoza A Michaels AJ Cols M. Temporal dynamics of persistent germinal centers and memory B cell differentiation following respiratory virus infection. Cell Rep (2021) 37(6):109961. doi: 10.1016/j.celrep.2021.109961 34758310 PMC7612942

[17] Pedreira CE Costa ES Lecrevisse Q van Dongen JJ Orfao A Consortium E. Overview of clinical flow cytometry data analysis: recent advances and future challenges. Trends Biotechnol (2013) 31(7):415–25. doi: 10.1016/j.tibtech.2013.04.008 23746659

[18] Rickinson A Kieff E. Epstein-Barr virus. Philadelphia: Fields Virology, Lippincott Williams, & Wilkins (2007), 2655–700 p.

[19] Nemerow GR McNaughton ME Cooper NR. Binding of monoclonal antibody to the Epstein Barr virus (EBV)/CR2 receptor induces activation and differentiation of human B lymphocytes. J Immunol (1985) 135(5):3068–73. 2995485

[20] Tanner J Weis J Fearon D Whang Y Kieff E. Epstein-Barr virus gp350/220 binding to the B lymphocyte C3d receptor mediates adsorption, capping, and endocytosis. Cell (1987) 50(2):203–13. doi: 10.1016/0092-8674(87)90216-9 3036369

[21] Fingeroth JD Weis JJ Tedder TF Strominger JL Biro PA Fearon DT. Epstein-Barr virus receptor of human B lymphocytes is the C3d receptor CR2. Proc Natl Acad Sci (1984) 81(14):4510–4. doi: 10.1073/pnas.81.14.4510 PMC345620 6087328

[22] Lindahl T Adams A Bjursell G Bornkamm GW Kaschka-Dierich C Jehn U. Covalently closed circular duplex DNA of Epstein-Barr virus in a human lymphoid cell line. J Mol Biol (1976) 102(3):511–30. doi: 10.1016/0022-2836(76)90331-4 178878

[23] Nonoyama M Pagano JS. Separation of Epstein-Barr virus DNA from large chromosomal DNA in non-virus-producing cells. Nat New Biol (1972) 238(84):169–71. doi: 10.1038/newbio238169a0 4340572

[24] Price AM Luftig MA. To be or not IIb: a multi-step process for Epstein-Barr virus latency establishment and consequences for B cell tumorigenesis. PloS Pathogens (2015) 11(3):e1004656. doi: 10.1371/journal.ppat.1004656 25790223 PMC4366242

[25] Lu F Chen H-S Kossenkov AV DeWispeleare K Won K-J Lieberman PM. EBNA2 drives formation of new chromosome binding sites and target genes for B-cell master regulatory transcription factors RBP-jκ and EBF1. PloS Pathogens (2016) 12(1):e1005339. doi: 10.1371/journal.ppat.1005339 26752713 PMC4709166

[26] Spender LC Cornish GH Sullivan A Farrell PJ. Expression of transcription factor AML-2 (RUNX3, CBF(alpha)-3) is induced by Epstein-Barr virus EBNA-2 and correlates with the B-cell activation phenotype. J Virol (2002) 76(10):4919–27. doi: 10.1128/jvi.76.10.4919-4927.2002 PMC136164 11967309

[27] Harada S Kieff E. Epstein-Barr virus nuclear protein LP stimulates EBNA-2 acidic domain-mediated transcriptional activation. J Virol (1997) 71(9):6611–8. doi: 10.1128/jvi.76.9.6611-6618.1997 PMC191939 9261383

[28] Sinclair AJ Palmero I Peters G Farrell P. EBNA-2 and EBNA-LP cooperate to cause G0 to G1 transition during immortalization of resting human B lymphocytes by Epstein-Barr virus. EMBO J (1994) 13(14):3321–8. doi: 10.1002/j.1460-2075.1994.tb06634.x/ PMC395229 8045261

[29] Mannick J Cohen J Birkenbach M Marchini A Kieff E. The Epstein-Barr virus nuclear protein encoded by the leader of the EBNA RNAs is important in B-lymphocyte transformation. J Virol (1991) 65(12):6826–37. doi: 10.1128/jvi.65.12.6826-6837.1991 PMC250776 1658376

[30] Sung N Kenney S Gutsch D Pagano J. EBNA-2 transactivates a lymphoid-specific enhancer in the BamHI C promoter of Epstein-Barr virus. J Virol (1991) 65(5):2164–9. doi: 10.1128/jvi.65.5.2164-2169.1991 PMC240563 1850003

[31] Hung SC Kang MS Kieff E. Maintenance of Epstein-Barr virus (EBV) oriP-based episomes requires EBV-encoded nuclear antigen-1 chromosome-binding domains, which can be replaced by high-mobility group-I or histone H1. Proc Natl Acad Sci USA (2001) 98(4):1865–70. doi: 10.1073/pnas.98.4.1865 PMC29348 11172042

[32] Price AM Dai J Bazot Q Patel L Nikitin PA Djavadian R. Epstein-Barr virus ensures B cell survival by uniquely modulating apoptosis at early and late times after infection. Elife (2017) 6:e22509. doi: 10.7554/eLife.22509 28425914 PMC5425254

[33] Kalchschmidt JS Gillman AC Paschos K Bazot Q Kempkes B Allday MJ. EBNA3C directs recruitment of RBPJ (CBF1) to chromatin during the process of gene repression in EBV infected B cells. PloS Pathogens (2016) 12(1):e1005383. doi: 10.1371/journal.ppat.1005383 26751214 PMC4708995

[34] Allday MJ Bazot Q White RE. The EBNA3 family: two oncoproteins and a tumour suppressor that are central to the biology of EBV in B cells. Epstein Barr Virus (2015) 2:61–117. doi: 10.1007/978-3-319-22834-1_3 26428372

[35] Wang A Welch R Zhao B Ta T Keleş S Johannsen E. Epstein-Barr virus nuclear antigen 3 (EBNA3) proteins regulate EBNA2 binding to distinct RBPJ genomic sites. J Virol (2015) 90(6):2906–19. doi: 10.1128/jvi.02737-15 PMC4810642 26719268

[36] Paschos K Parker GA Watanatanasup E White RE Allday MJ. BIM promoter directly targeted by EBNA3C in polycomb-mediated repression by EBV. Nucleic Acids Res (2012) 40(15):7233–46. doi: 10.1093/nar/gks391 PMC3424555 22584624

[37] White RE Groves IJ Turro E Yee J Kremmer E Allday MJ. Extensive co-operation between the Epstein-Barr virus EBNA3 proteins in the manipulation of host gene expression and epigenetic chromatin modification. PloS One (2010) 5(11):e13979. doi: 10.1371/journal.pone.0013979 21085583 PMC2981562

[38] Skalska L White RE Franz M Ruhmann M Allday MJ. Epigenetic repression of p16INK4A by latent Epstein-Barr virus requires the interaction of EBNA3A and EBNA3C with CtBP. PloS Pathogens (2010) 6(6):e1000951. doi: 10.1371/journal.ppat.1000951 20548956 PMC2883600

[39] Paschos K Smith P Anderton E Middeldorp JM White RE Allday MJ. Epstein-barr virus latency in B cells leads to epigenetic repression and CpG methylation of the tumour suppressor gene Bim. PloS Pathogens (2009) 5(6):e1000492. doi: 10.1371/journal.ppat.1000492 19557159 PMC2695769

[40] Radkov SA Bain M Farrell PJ West M Rowe M Allday MJ. Epstein-Barr virus EBNA3C represses Cp, the major promoter for EBNA expression, but has no effect on the promoter of the cell gene CD21. J Virol (1997) 71(11):8552–62. doi: 10.1128/jvi.71.11.8552-8562.1997 PMC192319 9343213

[41] Roughan JE Torgbor C Thorley-Lawson DA. Germinal center B cells latently infected with Epstein-Barr virus proliferate extensively but do not increase in number. J Virol (2010) 84(2):1158–68. doi: 10.1128/jvi.01780-09 PMC2798379 19889783

[42] Roughan JE Thorley-Lawson DA. The intersection of Epstein-Barr virus with the germinal center. J Virol (2009) 83(8):3968–76. doi: 10.1128/jvi.02609-08 PMC2663245 19193789

[43] Price AM Tourigny JP Forte E Salinas RE Dave SS Luftig MA. Analysis of Epstein-Barr virus-regulated host gene expression changes through primary B-cell outgrowth reveals delayed kinetics of latent membrane protein 1-mediated NF-κB activation. J Virol (2012) 86(20):11096–106. doi: 10.1128/jvi.01069-12 PMC3457162 22855490

[44] Basso K Dalla-Favera R. Germinal centres and B cell lymphomagenesis. Nat Rev Immunol (2015) 15(3):172–84. doi: 10.1038/nri3814 25712152

[45] Tsang S Wang F Izumi KM Kieff E. Delineation of the cis-acting element mediating EBNA-2 transactivation of latent infection membrane protein expression. J Virol (1991) 65(12):6765–71. doi: 10.1128/jvi.65.12.6765-6771.1991 PMC250762 1658373

[46] Wang F Tsang S Kurilla MG Cohen J Kieff E. Epstein-Barr virus nuclear antigen 2 transactivates latent membrane protein LMP1. J Virol (1990) 64(7):3407–16. doi: 10.1128/jvi.64.7.3407-3416.1990 PMC249594 2352328

[47] Cahir-McFarland ED Carter K Rosenwald A Giltnane JM Henrickson SE Staudt LM. Role of NF-κB in cell survival and transcription of latent membrane protein 1-expressing or Epstein-Barr virus latency III-infected cells. J Virol (2004) 78(8):4108–19. doi: 10.1128/jvi.78.8.4108-4119.2004 PMC374271 15047827

[48] Luftig M Prinarakis E Yasui T Tsichritzis T Cahir-McFarland E Inoue J-I. Epstein–Barr virus latent membrane protein 1 activation of NF-κB through IRAK1 and TRAF6. Proc Natl Acad Sci (2003) 100(26):15595–600. doi: 10.1073/pnas.2136756100 PMC307613 14673102

[49] Cahir-McFarland ED Davidson DM Schauer SL Duong J Kieff E. NF-κB inhibition causes spontaneous apoptosis in Epstein–Barr virus-transformed lymphoblastoid cells. Proc Natl Acad Sci (2000) 97(11):6055–60. doi: 10.1073/pnas.100119497 PMC18557 10811897

[50] Devergne O McFarland EC Mosialos G Izumi KM Ware CF Kieff E. Role of the TRAF binding site and NF-κB activation in Epstein-Barr virus latent membrane protein 1-induced cell gene expression. J Virol (1998) 72(10):7900–8. doi: 10.1128/jvi.72.10.7900-7908.1998 PMC110117 9733827

[51] Fish K Comoglio F Shaffer AL Ji Y Pan K-T Scheich S. Rewiring of B cell receptor signaling by Epstein–Barr virus LMP2A. Proc Natl Acad Sci (2020) 117(42):26318–27. doi: 10.1073/pnas.2007946117 PMC7584892 33020271

[52] Minamitani T Yasui T Ma Y Zhou H Okuzaki D Tsai C-Y. Evasion of affinity-based selection in germinal centers by Epstein–Barr virus LMP2A. Proc Natl Acad Sci (2015) 112(37):11612–7. doi: 10.1073/pnas.1514484112 PMC4577157 26305967

[53] Rancan C Schirrmann L Hüls C Zeidler R Moosmann A. Latent membrane protein LMP2A impairs recognition of EBV-infected cells by CD8+ T cells. PloS Pathogens (2015) 11(6):e1004906. doi: 10.1371/journal.ppat.1004906 26067064 PMC4465838

[54] Anderson LJ Longnecker R. EBV LMP2A provides a surrogate pre-B cell receptor signal through constitutive activation of the ERK/MAPK pathway. J Gen Virol (2008) 89. doi: 10.1099/vir.0.2008/001461-0 PMC2841786 18559925

[55] Portis T Longnecker R. Epstein–Barr virus (EBV) LMP2A mediates B-lymphocyte survival through constitutive activation of the Ras/PI3K/Akt pathway. Oncogene (2004) 23(53):8619–28. doi: 10.1038/sj.onc.1207905 15361852

[56] Thorley-Lawson DA. Epstein-Barr virus: exploiting the immune system. Nat Rev Immunol (2001) 1(1):75–82. doi: 10.1038/35095584 11905817

[57] Babcock GJ Decker LL Volk M Thorley-Lawson DA. EBV persistence in memory B cells in vivo. Immunity (1998) 9(3):395–404. doi: 10.1016/S1074-7613(00)80622-6 9768759

[58] Miyashita EM Yang B Babcock GJ Thorley-Lawson DA. Identification of the site of Epstein-Barr virus persistence *in vivo* as a resting B cell. J Virol (1997) 71(7):4882–91. doi: 10.1128/jvi.71.7.4882-4891.1997 PMC191718 9188550

[59] Styles CT Bazot Q Parker GA White RE Paschos K Allday MJ. EBV epigenetically suppresses the B cell-to-plasma cell differentiation pathway while establishing long-term latency. PloS Biol (2017) 15(8):e2001992. doi: 10.1371/journal.pbio.2001992 28771465 PMC5542390

[60] Laichalk LL Thorley-Lawson DA. Terminal differentiation into plasma cells initiates the replicative cycle of Epstein-Barr virus in vivo. J Virol (2005) 79(2):1296–307. doi: 10.1128/jvi.79.2.1296-1307.2005 PMC538585 15613356

[61] Schaeffner M Mrozek-Gorska P Buschle A Woellmer A Tagawa T Cernilogar FM. BZLF1 interacts with chromatin remodelers promoting escape from latent infections with EBV. Life Sci Alliance (2019) 2(2):e201800108. doi: 10.26508/lsa.201800108 30926617 PMC6441497

[62] Bhende PM Dickerson SJ Sun X Feng WH Kenney SC. X-box-binding protein 1 activates lytic Epstein-Barr virus gene expression in combination with protein kinase D. J Virol (2007) 81(14):7363–70. doi: 10.1128/jvi.00154-07 PMC1933364 17494074

[63] Sun CC Thorley-Lawson DA. Plasma cell-specific transcription factor XBP-1s binds to and transactivates the Epstein-Barr virus BZLF1 promoter. J Virol (2007) 81(24):13566–77. doi: 10.1128/jvi.01055-07 PMC2168822 17898050

[64] Speck SH Chatila T Flemington E. Reactivation of Epstein-Barr virus: regulation and function of the BZLF1 gene. Trends Microbiol (1997) 5(10):399–405. doi: 10.1016/S0966-842X(97)01129-3 9351176

[65] Thorley-Lawson DA Gross A. Persistence of the Epstein–Barr virus and the origins of associated lymphomas. New Engl J Med (2004) 350(13):1328–37. doi: 10.1056/NEJMra032015 15044644

[66] Niedobitek G Meru N Delecluse HJ. Epstein-Barr virus infection and human malignancies. Int J Exp Pathol (2001) 82(3):149–70. doi: 10.1111/j.1365-2613.2001.iep190.x PMC2517709 11488990

[67] Epstein M Achong B Barr Y. Virus particles in cultured lymphoblasts from Burkitt’s lymphoma. Lancet (1964) 283(7335):702–3. doi: 10.1016/S0140-6736(64)91524-7 14107961

[68] Zur Hausen H Schulte-Holthausen H Klein G Henle G Henle W Clifford P. Epstein-Barr virus in Burkitt's lymphoma and nasopharyngeal carcinoma. [ii] EBV DNA biopsies Burkitt tumours anaplastic carcinomas nasopharynx. Nat (1970) 228:1056–8. doi: 10.1038/2281056a0 4320657

[69] Moormann AM Snider CJ Chelimo K. The company malaria keeps: how co-infection with Epstein-Barr virus leads to endemic Burkitt lymphoma. Curr Opin Infect Dis (2011) 24(5):435. doi: 10.1097/QCO.0b013e328349ac4f 21885920 PMC3265160

[70] Niedobitek G Agathanggelou A Rowe M Jones E Jones D Turyaguma P. Heterogeneous expression of Epstein-Barr virus latent proteins in endemic Burkitt's lymphoma. Blood J Am Soc Hematol (1995) 82(2):659–65. doi: 10.1182/blood.V86.2.659.bloodjournal862659 7605996

[71] Carbone A Gloghini A Larocca LM Antinori A Falini B Tirelli U. Human immunodeficiency virus–associated Hodgkin’s disease derives from post–germinal center B cells. Blood J Am Soc Hematol (1999) 93(7):2319–26. doi: 10.1182/blood.V93.7.2319 10090942

[72] Cohen JI. Epstein–Barr virus infection. New Engl J Med (2000) 343(7):481–92. doi: 10.1056/NEJM200008173430707 10944566

[73] Carbone A Cesarman E Spina M Gloghini A Schulz TF. HIV-associated lymphomas and gamma-herpesviruses. Blood J Am Soc Hematol (2009) 113(6):1213–24. doi: 10.1182/blood-2008-09-180315 18955561

[74] Grogg K Miller R Dogan A. HIV infection and lymphoma. J Clin Pathol (2007) 60(12):1365–72. doi: 10.1136/jcp.2007.051953 PMC2095580 18042692

[75] Paya CV Fung JJ Nalesnik MA Kieff E Green M Gores G. Epstein-Barr virus-induced posttransplant lymphoproliferative disorders. Transplantation (1999) 68(10):1517–25. 10.1097/00007890-199911270-00015 10589949

[76] Rowe M Young L Crocker J Stokes H Henderson S Rickinson A. Epstein-Barr virus (EBV)-associated lymphoproliferative disease in the SCID mouse model: implications for the pathogenesis of EBV-positive lymphomas in man. J Exp Med (1991) 173(1):147–58. doi: 10.1084/jem.173.1.147 PMC2118756 1845872

[77] Cohen JI Fauci AS Varmus H Nabel GJ. Epstein-Barr virus: an important vaccine target for cancer prevention. Sci Trans Med (2011) 3(107):107fs7–fs7. doi: 10.1126/scitranslmed.3002878 PMC3501269 22049067

[78] Young LS Rickinson AB. Epstein-Barr virus: 40 years on. Nat Rev Cancer (2004) 4(10):757–68. doi: 10.1038/nrc1452 15510157

[79] Sun K Jia K Lv H Wang S-Q Wu Y Lei H. EBV-positive gastric cancer: current knowledge and future perspectives. Front Oncol (2020) 10:583463. doi: 10.3389/fonc.2020.583463 33381453 PMC7769310

[80] Larsen M Sauce D Deback C Arnaud L Mathian A Miyara M. Exhausted cytotoxic control of Epstein-Barr virus in human lupus. PloS Pathogens (2011) 7(10):e1002328. doi: 10.1371/journal.ppat.1002328 22028659 PMC3197610

[81] Poole BD Templeton AK Guthridge JM Brown EJ Harley JB James JA. Aberrant Epstein–Barr viral infection in systemic lupus erythematosus. Autoimmun Rev (2009) 8(4):337–42. doi: 10.1016/j.autrev.2008.12.008 PMC2822456 19167523

[82] Gross AJ Hochberg D Rand WM Thorley-Lawson DA. EBV and systemic lupus erythematosus: a new perspective. J Immunol (2005) 174(11):6599–607. doi: 10.4049/jimmunol.174.11.6599 15905498

[83] Kang I Quan T Nolasco H Park S-H Hong MS Crouch J. Defective control of latent Epstein-Barr virus infection in systemic lupus erythematosus. J Immunol (2004) 172(2):1287–94. doi: 10.4049/jimmunol.172.2.1287 14707107

[84] Tsokos G Magrath I Balow J. Epstein-Barr virus induces normal B cell responses but defective suppressor T cell responses in patients with systemic lupus erythematosus. J Immunol (1983) 131(4):1797–801. 6311898

[85] Mouat IC Morse ZJ Shanina I Brown KL Horwitz MS. Latent gammaherpesvirus exacerbates arthritis through modification of age-associated B cells. Elife (2021) 10:e67024. doi: 10.7554/eLife.67024 34080972 PMC8337075

[86] Balandraud N Roudier J Roudier C. Epstein–Barr virus and rheumatoid arthritis. Autoimmun Rev (2004) 3(5):362–7. doi: 10.1016/j.autrev.2004.02.002 15288002

[87] Balandraud N Meynard JB Auger I Sovran H Mugnier B Reviron D. Epstein-Barr virus load in the peripheral blood of patients with rheumatoid arthritis: accurate quantification using real-time polymerase chain reaction. Arthritis Rheumatism (2003) 48(5):1223–8. doi: 10.1002/art.10933 12746895

[88] Tosato G Steinberg A Yarchoan R Heilman C Pike S De Seau V. Abnormally elevated frequency of Epstein-Barr virus-infected B cells in the blood of patients with rheumatoid arthritis. J Clin Invest (1984) 73(6):1789–95. doi: 10.1172/JCI111388 PMC437092 6327772

[89] Tosato G Steinberg AD Blaese RM. Defective EBV-specific suppressor T-cell function in rheumatoid arthritis. New Engl J Med (1981) 305(21):1238–43. doi: 10.1056/NEJM198111193052102 6270556

[90] Cavalcante P Galbardi B Franzi S Marcuzzo S Barzago C Bonanno S. Increased expression of Toll-like receptors 7 and 9 in myasthenia gravis thymus characterized by active Epstein–Barr virus infection. Immunobiology (2016) 221(4):516–27. doi: 10.1016/j.imbio.2015.12.007 26723518

[91] Saito I Servenius B Compton T Fox RI. Detection of Epstein-Barr virus DNA by polymerase chain reaction in blood and tissue biopsies from patients with Sjogren's syndrome. J Exp Med (1989) 169(6):2191–8. doi: 10.1084/jem.169.6.2191 PMC2189340 2543732

[92] Fox R Pearson G Vaughan J. Detection of Epstein-Barr virus-associated antigens and DNA in salivary gland biopsies from patients with Sjogren's syndrome. J Immunol (1986) 137(10):3162–8. 3021847

[93] Rickinson A. editor Co-infections, inflammation and oncogenesis: Future directions for EBV research. Semin Cancer Biol (2014) 26:99–115. doi: 10.1016/j.semcancer.2014.04.004 24751797

[94] Ascherio A Munger KL. EBV and autoimmunity. Epstein Barr Virus 1:365–85. doi: 10.1007/978-3-319-22822-8_15 26424654

[95] Ascherio A Munger KL Lennette ET Spiegelman D Hernán MA Olek MJ. Epstein-Barr virus antibodies and risk of multiple sclerosis: a prospective study. JAMA (2001) 286(24):3083–8. doi: 10.1001/jama.286.24.3083 11754673

[96] Bjornevik K Cortese M Healy BC Kuhle J Mina MJ Leng Y. Longitudinal analysis reveals high prevalence of Epstein-Barr virus associated with multiple sclerosis. Science (2022) 375(6578):296–301. doi: 10.1126/science.abj8222 35025605

[97] Lanz TV Brewer RC Ho PP Moon J-S Jude KM Fernandez D. Clonally Expanded B Cells in Multiple Sclerosis Bind EBV EBNA1 and GlialCAM. Nature (2022) 603(7900):321–7. doi: 10.1038/241586-022-04432-7 PMC9382663 35073561

[98] Karnell JL Kumar V Wang J Wang S Voynova E Ettinger R. Role of CD11c+ T-bet+ B cells in human health and disease. Cell Immunol (2017) 321:40–5. doi: 10.1016/j.cellimm.2017.05.008 28756897

[99] Naradikian MS Myles A Beiting DP Roberts KJ Dawson L Herati RS. Cutting edge: IL-4, IL-21, and IFN-γ interact to govern T-bet and CD11c expression in TLR-activated B cells. J Immunol (2016) 197(4):1023–8. doi: 10.4049/jimmunol.1600522 PMC4975960 27430719

[100] Rubtsov AV Rubtsova K Fischer A Meehan RT Gillis JZ Kappler JW. Toll-like receptor 7 (TLR7)–driven accumulation of a novel CD11c+ B-cell population is important for the development of autoimmunity. Blood J Am Soc Hematol (2011) 118(5):1305–15. doi: 10.1182/blood-2011-01-331462 PMC3152497 21543762

[101] Johnson JL Rosenthal RL Knox JJ Myles A Naradikian MS Madej J. The transcription factor T-bet resolves memory B cell subsets with distinct tissue distributions and antibody specificities in mice and humans. Immunity (2020) 52(5):842–55. doi: 10.1016/j.immuni.2020.03.020 PMC7242168 32353250

[102] Austin JW Buckner CM Kardava L Wang W Zhang X Melson VA. Overexpression of T-bet in HIV infection is associated with accumulation of B cells outside germinal centers and poor affinity maturation. Sci Trans Med (2019) 11(520):eaax0904. doi: 10.1126/scitranslmed.aax0904 PMC7479651 31776286

[103] Rubtsova K Rubtsov AV Thurman JM Mennona JM Kappler JW Marrack P. B cells expressing the transcription factor T-bet drive lupus-like autoimmunity. J Clin Invest (2017) 127(4):1392–404. doi: 10.1172/JCI91250 PMC5373868 28240602

[104] Rubtsova K Marrack P Rubtsov AV. TLR7, IFNγ, and T-bet: Their roles in the development of ABCs in female-biased autoimmunity. Cell Immunol (2015) 294(2):80–3. doi: 10.1016/j.cellimm.2014.12.002 PMC4380581 25541140

[105] Weiss GE Crompton PD Li S Walsh LA Moir S Traore B. Atypical memory B cells are greatly expanded in individuals living in a malaria-endemic area. J Immunol (2009) 183(3):2176–82. doi: 10.4049/jimmunol.0901297 PMC2713793 19592645

[106] Moir S Ho J Malaspina A Wang W DiPoto AC O'Shea MA. Evidence for HIV-associated B cell exhaustion in a dysfunctional memory B cell compartment in HIV-infected viremic individuals. J Exp Med (2008) 205(8):1797–805. doi: 10.1084/jem.20072683 PMC2525604 18625747

[107] Hafez AY Luftig MA. Characterization of the EBV-induced persistent DNA damage response. Viruses (2017) 9(12):366. doi: 10.3390/v9120366 PMC5744141 29194355

[108] McFadden K Hafez AY Kishton R Messinger JE Nikitin PA Rathmell JC. Metabolic stress is a barrier to Epstein–Barr virus-mediated B-cell immortalization. Proc Natl Acad Sci (2016) 113(6):E782–E90. doi: 10.1073/pnas.151714113 PMC4760815 26802124

[109] Nikitin PA Price AM McFadden K Yan CM Luftig MA. Mitogen-induced B-cell proliferation activates Chk2-dependent G1/S cell cycle arrest. PloS One (2014) 9(1):e87299. doi: 10.1371/journal.pone.0087299 24498068 PMC3907503

[110] Forte E Luftig MA. MDM2-dependent inhibition of p53 is required for Epstein-Barr virus B-cell growth transformation and infected-cell survival. J Virol (2009) 83(6):2491–9. doi: 10.1128/jvi.01681-08 PMC2648290 19144715

[111] SoRelle ED Dai J Bonglack EN Heckenberg EM Zhou JY Giamberardino SN. Single-cell RNA-seq reveals transcriptomic heterogeneity mediated by host–pathogen dynamics in lymphoblastoid cell lines. Elife (2021) 10:e62586. doi: 10.7554/eLife.62586 33501914 PMC7867410

[112] Osorio D Yu X Yu P Serpedin E Cai JJ. Single-cell RNA sequencing of a European and an African lymphoblastoid cell line. Sci Data (2019) 6(1):112. doi: 10.1038/s41597-019-0116-4 31273215 PMC6609777

[113] Stuart T Butler A Hoffman P Hafemeister C Papalexi E Mauck WM III. Comprehensive integration of single-cell data. Cell (2019) 177(7):1888–902:e21. doi: 10.1016/j.cell.2019.05.031 PMC6687398 31178118

[114] Satija R Farrell JA Gennert D Schier AF Regev A. Spatial reconstruction of single-cell gene expression data. Nat Biotechnol (2015) 33(5):495–502. doi: 10.1038/nbt.3192 25867923 PMC4430369

[115] Hao Y Hao S Andersen-Nissen E Mauck WM III Zheng S Butler A. Integrated analysis of multimodal single-cell data. Cell (2021) 184(13):3573–87. doi: 10.1016/j.cell.2021.04.048 PMC8238499 34062119

[116] McInnes L Healy J Melville J. Umap: Uniform manifold approximation and projection for dimension reduction. (2018), 18020342. doi: 10.48550/arXic.1802.03426

[117] Linderman GC Zhao J Roulis M Bielecki P Flavell RA Nadler B. Zero-preserving imputation of single-cell RNA-seq data. Nat Commun (2022) 13(1):1–11. doi: 10.1038/s41467-021-27729-z 35017482 PMC8752663

[118] Qiu X Mao Q Tang Y Wang L Chawla R Pliner HA. Reversed graph embedding resolves complex single-cell trajectories. Nat Methods (2017) 14(10):979. doi: 10.1038/nmeth.4402 28825705 PMC5764547

[119] Trapnell C. Defining cell types and states with single-cell genomics. Genome Res (2015) 25(10):1491–8. doi: 10.1101/gr.190595.115 PMC4579334 26430159

[120] Trapnell C Cacchiarelli D Grimsby J Pokharel P Li S Morse M. The dynamics and regulators of cell fate decisions are revealed by pseudotemporal ordering of single cells. Nat Biotechnol (2014) 32(4):381. doi: 10.1038/nbt.2859 24658644 PMC4122333

[121] Nichele I Zamo A Bertolaso A Bifari F Tinelli M Franchini M. VR09 cell line: an EBV-positive lymphoblastoid cell line with *in vivo* characteristics of diffuse large B cell lymphoma of activated B-cell type. PloS One (2012) 7(12):e52811. doi: 10.1371/journal.pone.0052811 23285191 PMC3528718

[122] Messinger JE Dai J Stanland LJ Price AM Luftig MA. Identification of Host Biomarkers of Epstein-Barr Virus Latency IIb and Latency III. mBio (2019) 10(4):e01006–19. doi: 10.1128/mBio.01006-19 PMC6606803 31266868

[123] Mesin L Ersching J Victora GD. Germinal center B cell dynamics. Immunity (2016) 45(3):471–82. doi: 10.1016/j.immuni.2016.09.001 PMC5123673 27653600

[124] Suan D Kräutler NJ Maag JL Butt D Bourne K Hermes JR. CCR6 defines memory B cell precursors in mouse and human germinal centers, revealing light-zone location and predominant low antigen affinity. Immunity (2017) 47(6):1142–53. doi: 10.1016/j.immuni.2017.11.022 29262350

[125] Alizadeh AA Eisen MB Davis RE Ma C Lossos IS Rosenwald A. Distinct types of diffuse large B-cell lymphoma identified by gene expression profiling. Nature (2000) 403(6769):503–11. doi: 10.1038/35000501 10676951

[126] Wright G Tan B Rosenwald A Hurt EH Wiestner A Staudt LM. A gene expression-based method to diagnose clinically distinct subgroups of diffuse large B cell lymphoma. Proc Natl Acad Sci (2003) 100(17):9991–6. doi: 10.1073/pnas.1732008100 PMC187912 12900505

[127] Klein U Tu Y Stolovitzky GA Keller JL Haddad J Miljkovic V. Transcriptional analysis of the B cell germinal center reaction. Proc Natl Acad Sci (2003) 100(5):2639–44. doi: 10.1073/pnas.0437996100 PMC151393 12604779

[128] Montes-Moreno S Roncador G Maestre L Martinez N Sanchez-Verde L Camacho FI. Gcet1 (centerin), a highly restricted marker for a subset of germinal center-derived lymphomas. Blood J Am Soc Hematol (2008) 111(1):351–8. doi: 10.1182/blood-2007-06-094151 17898315

[129] Nagashima T Ichimiya S Kikuchi T Saito Y Matsumiya H Ara S. Arachidonate 5-lipoxygenase establishes adaptive humoral immunity by controlling primary B cells and their cognate T-cell help. Am J Pathol (2011) 178(1):222–32. doi: 10.1016/j.ajpath/2010.11.033 PMC3070564 21224059

[130] Basso K Schneider C Shen Q Holmes AB Setty M Leslie C. BCL6 positively regulates AID and germinal center gene expression *via* repression of miR-155. J Exp Med (2012) 209(13):2455–65. doi: 10.1084/jem.20121387 PMC3526356 23166356

[131] Basso K Dalla-Favera R. BCL6: master regulator of the germinal center reaction and key oncogene in B cell lymphomagenesis. Adv Immunol (2010) 105:193–210. doi: 10.1016/S0065-2776(10)05007-8 20510734

[132] Muppidi JR Schmitz R Green JA Xiao W Larsen AB Braun SE. Loss of signalling *via* Gα13 in germinal centre B-cell-derived lymphoma. Nature (2014) 516(7530):254–8. doi: 10.1038/nature13765 PMC4267955 25274307

[133] Pan Z Shen Y Du C Zhou G Rosenwald A Staudt LM. Two newly characterized germinal center B-cell-associated genes, GCET1 and GCET2, have differential expression in normal and neoplastic B cells. Am J Pathol (2003) 163(1):135–44. doi: 10.1016/S0002-9440(10)63637-1 PMC1868163 12819018

[134] Radtke D Bannard O. Expression of the plasma cell transcriptional regulator Blimp-1 by dark zone germinal center B cells during periods of proliferation. Front Immunol (2019) 9:3106. doi: 10.3389/fimmu.2018.03106 30687317 PMC6334666

[135] Sander S Yasuda T Franklin A Graf R Calado DP Li S. PI3 kinase and FOXO1 transcription factor activity differentially control B cells in the germinal center light and dark zones. Immunity (2015) 43(6):1075–86. doi: 10.1016/j.immuni.2015.10.021 26620760

[136] Martin-Perez D Vargiu P Montes-Moreno S Leon E Rodriguez-Pinilla S Lisio L. Epstein-Barr virus microRNAs repress BCL6 expression in diffuse large B-cell lymphoma. Leukemia (2012) 26(1):180–3. doi: 10.1038/leu.2011.189 21788950

[137] Shi W Liao Y Willis SN Taubenheim N Inouye M Tarlinton DM. Transcriptional profiling of mouse B cell terminal differentiation defines a signature for antibody-secreting plasma cells. Nat Immunol (2015) 16(6):663–73. doi: 10.1038/ni.3154 25894659

[138] Minnich M Tagoh H Bönelt P Axelsson E Fischer M Cebolla B. Multifunctional role of the transcription factor Blimp-1 in coordinating plasma cell differentiation. Nat Immunol (2016) 17(3):331–43. doi: 10.1038/ni.3349 PMC5790184 26779602

[139] Morgan D Tergaonkar V. Unraveling B cell trajectories at single cell resolution. Trends Immunol (2022) 43(3):210–29. doi: 10.1016/j.it.2022.01.003 35090788

[140] Stein N Berhani O Schmiedel D Duev-Cohen A Seidel E Kol I. IFNG-AS1 enhances interferon gamma production in human natural killer cells. Iscience (2019) 11:466–73. doi: 10.1016/j.isci.2018.12.034 PMC6354656 30661002

[141] Price MJ Patterson DG Scharer CD Boss JM. Progressive upregulation of oxidative metabolism facilitates plasmablast differentiation to a T-independent antigen. Cell Rep (2018) 23(11):3152–9. doi: 10.1016/j.celrep.2018.05.053 PMC6092755 29898388

[142] Ahsan N Kanda T Nagashima K Takada K. Epstein-Barr virus transforming protein LMP1 plays a critical role in virus production. J Virol (2005) 79(7):4415–24. doi: 10.1128/jvi.79.7.4415-4424.2005 PMC1061545 15767441

[143] Yuan J Cahir-McFarland E Zhao B Kieff E. Virus and cell RNAs expressed during Epstein-Barr virus replication. J Virol (2006) 80(5):2548–65. doi: 10.1128/jvi.80.5.2548-2565.2006 PMC1395376 16474161

[144] Davies ML Xu S Lyons-Weiler J Rosendorff A Webber SA Wasil LR. Cellular factors associated with latency and spontaneous Epstein–Barr virus reactivation in B-lymphoblastoid cell lines. Virol (2010) 400(1):53–67. doi: 10.1016/j.virol.2010.01.002 20153012

[145] Jenks SA Cashman KS Zumaquero E Marigorta UM Patel AV Wang X. Distinct effector B cells induced by unregulated toll-like receptor 7 contribute to pathogenic responses in systemic lupus erythematosus. Immunity (2018) 49(4):725–39:e6. doi: 10.1016/j.immuni.2018.08.015 PMC6217820 30314758

[146] Yeo L Lom H Juarez M Snow M Buckley C Filer A. Expression of FcRL4 defines a pro-inflammatory, RANKL-producing B cell subset in rheumatoid arthritis. Ann Rheumatic Dis (2015) 74(5):928–35. doi: 10.1136/annrheumdis-2013-204116 PMC4392201 24431391

[147] Li H Tolnay M. FCRL4 and FCRL5 expression distinguishes three human memory B cell subsets in tonsils. J Immunol (2017) 198(1):144.

[148] Sohn HW Krueger PD Davis RS Pierce SK. FcRL4 acts as an adaptive to innate molecular switch dampening BCR signaling and enhancing TLR signaling. Blood J Am Soc Hematol (2011) 118(24):6332–41. doi: 10.1182/blood-2011-05-353102 PMC3236118 21908428

[149] Zumaquero E Stone SL Scharer CD Jenks SA Nellore A Mousseau B. IFNγ induces epigenetic programming of human T-bethi B cells and promotes TLR7/8 and IL-21 induced differentiation. Elife (2019) 8:e41641. doi: 10.7554/eLife.41641 31090539 PMC6544433

[150] Stone SL Peel JN Scharer CD Risley CA Chisolm DA Schultz MD. T-bet transcription factor promotes antibody-secreting cell differentiation by limiting the inflammatory effects of IFN-γ on B cells. Immunity (2019) 50(5):1172–87. doi: 10.1016/j.immuni.2019.04.004 PMC6929688 31076359

[151] van Langelaar J Wierenga-Wolf AF Samijn JP Luijks CJ Siepman TA van Doorn PA. The association of Epstein-Barr virus infection with CXCR3+ B-cell development in multiple sclerosis: impact of immunotherapies. Eur J Immunol (2021) 51(3):626–33. doi: 10.1002/eji.202048739 PMC7984177 33152118

[152] van Langelaar J Rijvers L Janssen M Wierenga-Wolf AF Melief MJ Siepman TA. Induction of brain-infiltrating T-bet–expressing B cells in multiple sclerosis. Ann Neurol (2019) 86(2):264–78. doi: 10.1002/ana.25508 PMC6771938 31136008

[153] Leffler J Trend S Ward NC Grau GE Hawke S Byrne SN. Circulating memory B cells in early multiple sclerosis exhibit increased IgA+ cells, globally decreased BAFF-R expression and an EBV-related IgM+ cell signature. Front Immunol (2022) 525:812317. doi: 10.3389/fimmu.2022.812317 PMC8888440 35250986

[154] Thorley-Lawson DA Hawkins JB Tracy SI Shapiro M. The pathogenesis of Epstein–Barr virus persistent infection. Curr Opin Virol (2013) 3(3):227–32. doi: 10.1016/j.coviro.2013.04.005 PMC3789532 23683686

[155] Klein E Nagy N Rasul AE. EBV genome carrying B lymphocytes that express the nuclear protein EBNA-2 but not LMP-1: Type IIb latency. Oncoimmunology (2013) 2(2):e23035. doi: 10.4161/onci.23035 23526738 PMC3601171

[156] Jochum S Ruiss R Moosmann A Hammerschmidt W Zeidler R. RNAs in Epstein–Barr virions control early steps of infection. Proc Natl Acad Sci (2012) 109(21):E1396–404. doi: 10.1073/pnas.1115906109 PMC3361417 22543160

[157] Mrozek-Gorska P Buschle A Pich D Schwarzmayr T Fechtner R Scialdone A. Epstein-Barr virus reprograms human B lymphocytes immediately in the prelatent phase of infection. Proc Natl Acad Sci (2019) 116(32):16046–55. doi: 10.1073/pnas.1901314116 PMC6690029 31341086

[158] Bristol JA Brand J Ohashi M Eichelberg MR Casco A Nelson SE. Reduced IRF4 expression promotes lytic phenotype in Type 2 EBV-infected B cells. PloS Pathogens (2022) 18(4):e1010453. doi: 10.1371/journal.ppat.1010453 35472072 PMC9041801

[159] Boccellato F Anastasiadou E Rosato P Kempkes B Frati L Faggioni A. EBNA2 interferes with the germinal center phenotype by downregulating BCL6 and TCL1 in non-Hodgkin's lymphoma cells. J Virol (2007) 81(5):2274–82. doi: 10.1128/jvi.01822-06 PMC1865942 17151114

[160] Dai J Heckenberg E Song L Crawford GE Luftig MA. Epstein-Barr virus promotes survival through germinal center light zone chromatin architecture. bioRxiv (2020). doi: 10.1101/2020.10.22.350108 PMC10790771 38059622

[161] Naradikian MS Hao Y Cancro MP. Age-associated B cells: key mediators of both protective and autoreactive humoral responses. Immunol Rev (2016) 269(1):118–29. doi: 10.1111/imr.12380 26683149

[162] Rubtsova K Rubtsov AV Cancro MP Marrack P. Age-associated B cells: a T-bet–dependent effector with roles in protective and pathogenic immunity. J Immunol (2015) 195(5):1933–7. doi: 10.4049/jimmunol.1501209 PMC4548292 26297793

[163] Verstappen GM Ice JA Bootsma H Pringle S Haacke EA de Lange K. Gene expression profiling of epithelium-associated FcRL4+ B cells in primary Sjögren's syndrome reveals a pathogenic signature. J Autoimmun (2020) 109:102439. doi: 10.1016/j.aut.2020.102439 32201227 PMC7337041

[164] Xu J Neal LM Ganguly A Kolbe JL Hargarten JC Elsegeiny W. Chemokine receptor CXCR3 is required for lethal brain Pathology but not pathogen clearance during cryptococcal meningoencephalitis. Sci Adv (2020) 6(25):eaba2502. doi: 10.1126/sciadv.aba2502 32596454 PMC7299622

[165] Marques CP Kapil P Hinton DR Hindinger C Nutt SL Ransohoff RM. CXCR3-dependent plasma blast migration to the central nervous system during viral encephalomyelitis. J Virol (2011) 85(13):6136–47. doi: 10.1128/jvi.00202-11 PMC3126522 21507985

[166] Santamaria K Desmots F Leonard S Caron G Haas M Delaloy C. Committed Human CD23-Negative Light-Zone Germinal Center B Cells Delineate Transcriptional Program Supporting Plasma Cell Differentiation. Front Immunol (2021) 12:5142. doi: 10.3389/fimmu.2021.744573 PMC8674954 34925321

[167] Sanz I Wei C Jenks SA Cashman KS Tipton C Woodruff MC. Challenges and opportunities for consistent classification of human B cell and plasma cell populations. Front Immunol (2019) 10:2458. doi: 10.3389/fimmu.2019.02458 31681331 PMC6813733

[168] Scharer CD Blalock EL Mi T Barwick BG Jenks SA Deguchi T. Epigenetic programming underpins B cell dysfunction in human SLE. Nat Immunol (2019) 20(8):1071–82. doi: 10.1038/s41590-019-0419-9 PMC6642679 31263277

[169] Ambegaonkar AA Holla P Dizon BL Sohn H Pierce SK. Atypical B cells in chronic infectious Dis and systemic autoimmunity: puzzles with many missing pieces. Curr Opin Immunol (2022) 77:102227. doi: 10.1016/j.coi.2022.102227 35724448 PMC9612402

[170] Martin HJ Lee JM Walls D Hayward SD. Manipulation of the toll-like receptor 7 signaling pathway by Epstein-Barr virus. J Virol (2007) 81(18):9748–58. doi: 10.1128/jvi.01122-07 PMC2045431 17609264

