# Supplementary material for: Epstein-Barr virus perpetuates B cell germinal center dynamics and generation of autoimmune-associated phenotypes in vitro
Source: Front Immunol. 2022 Sep 28;13:1001145. doi: 10.3389/fimmu.2022.1001145 (PMC9554744; doi:10.3389/fimmu.2022.1001145)
Supplement: Supplementary Figure 1 — Additional LCL replicates for FACS populations and sorting. (A) Biological replicates for ICAM-1/CD27 FACS staining. (B) Phenotype composition in LCLs is dependent on cell density. (C) Biological replicates for time-resolved assays of ICAM-1 and CD27 in sorted LCL fractions. Top row in each replicate: ICAM-1Hi/CD27Lo. Middle row in each replicate: ICAM-1Lo/CD27Lo. Bottom row in each replicate: ICAM-1Lo/CD27Hi. The box inset depicts a technical artifact arising from FACS buffer contamination that affected the observed CD27/ICAM1 distribution from days 3-5. (D) Replicate experiments performed with increased gating stringency to exclude the possibility of phenotype contamination leading to the observed recovery of parental line distributions. [file DataSheet_1.pdf]

Supplementary Figures for

Epstein-Barr virus perpetuates B cell germinal center dynamics and generation of autoimmune-associated phenotypes *in vitro*

Elliott D. SoRelle[1,2,#,*] , Nicolás M. Reinoso-Vizcaino[1,#,*], Gillian Q. Horn[3], & Micah A. Luftig[1*]

[1]Department of Molecular Genetics & Microbiology, Duke University, Durham, NC, USA
[2]Department of Biostatistics & Bioinformatics, Duke University, Durham, NC, USA
[3]Department of Immunology, Duke University, Durham, NC, USA

[#] These authors share first authorship

*** Correspondence:**
Corresponding Authors
elliott.sorelle@duke.edu, nico.reinoso@duke.edu, micah.luftig@duke.edu

Keywords: Epstein-Barr virus, B cell, germinal center, single-cell, lymphoblastoid cells, autoimmunity, chronic infection, atypical memory B cells

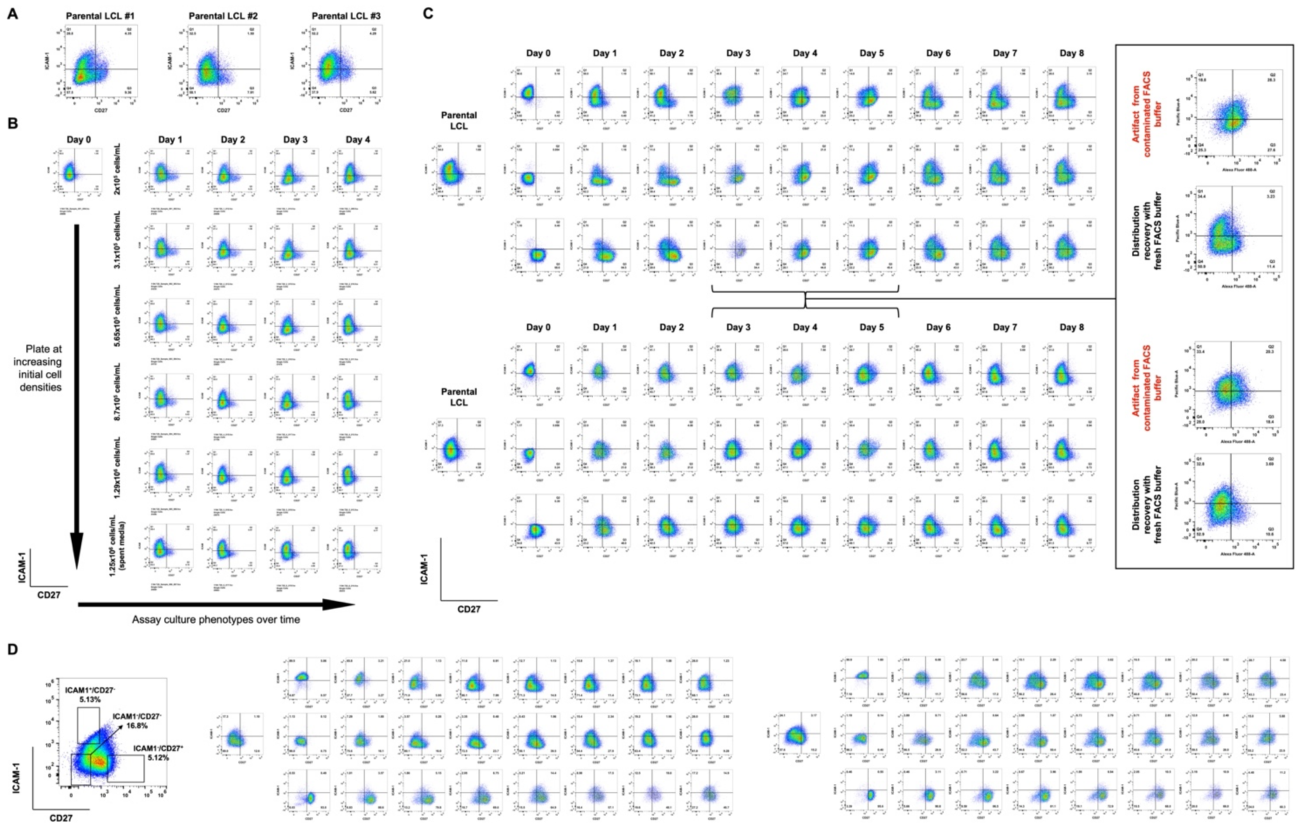

Supporting Figure 1. Additional LCL replicates for FACS populations and sorting.
(A) Biological replicates for ICAM-1/CD27 FACS staining.
(B) Phenotype composition in LCLs is dependent on cell density.
(C) Biological replicates for time-resolved assays of ICAM-1 and CD27 in sorted LCL fractions. Top row in each replicate: ICAM-1^{Hi}/$CD27^{Lo}$. Middle row in each replicate: ICAM-1^{Lo}/$CD27^{Lo}$. Bottom row in each replicate: ICAM-1^{Lo}/$CD27^{Hi}$. The box inset depicts a technical artifact arising from FACS buffer contamination that affected the observed CD27/ICAM1 distribution from days 3-5.
(D) Replicate experiments performed with increased gating stringency to exclude the possibility of phenotype contamination leading to the observed recovery of parental line distributions.

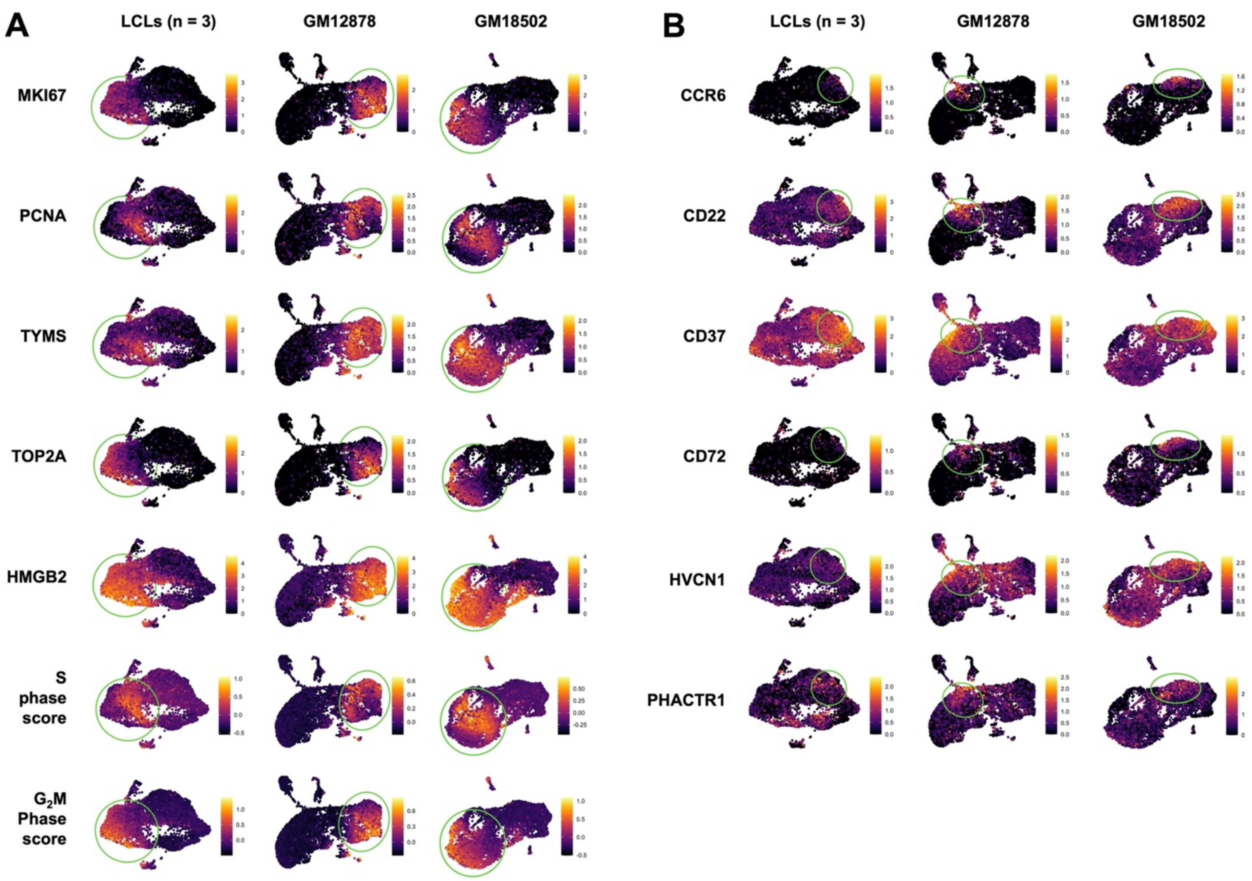

Supporting Figure 2. Additional marker genes in DZ and AP-eMBC states.
(A) Key marker genes in cycling (DZ-like) cells across LCLs. Data are presented for three LCLs generated in-house (left column) and commercially available LCLs (GM12878, middle; GM18502, right) originally reported by Osorio *et al*[1].
(B) Key marker genes in AP-eMBC cells across LCLs. Data are presented for three LCLs generated in-house (left column) and commercially available LCLs (GM12878, middle column; GM18502, right column) originally reported by Osorio *et al*[1].

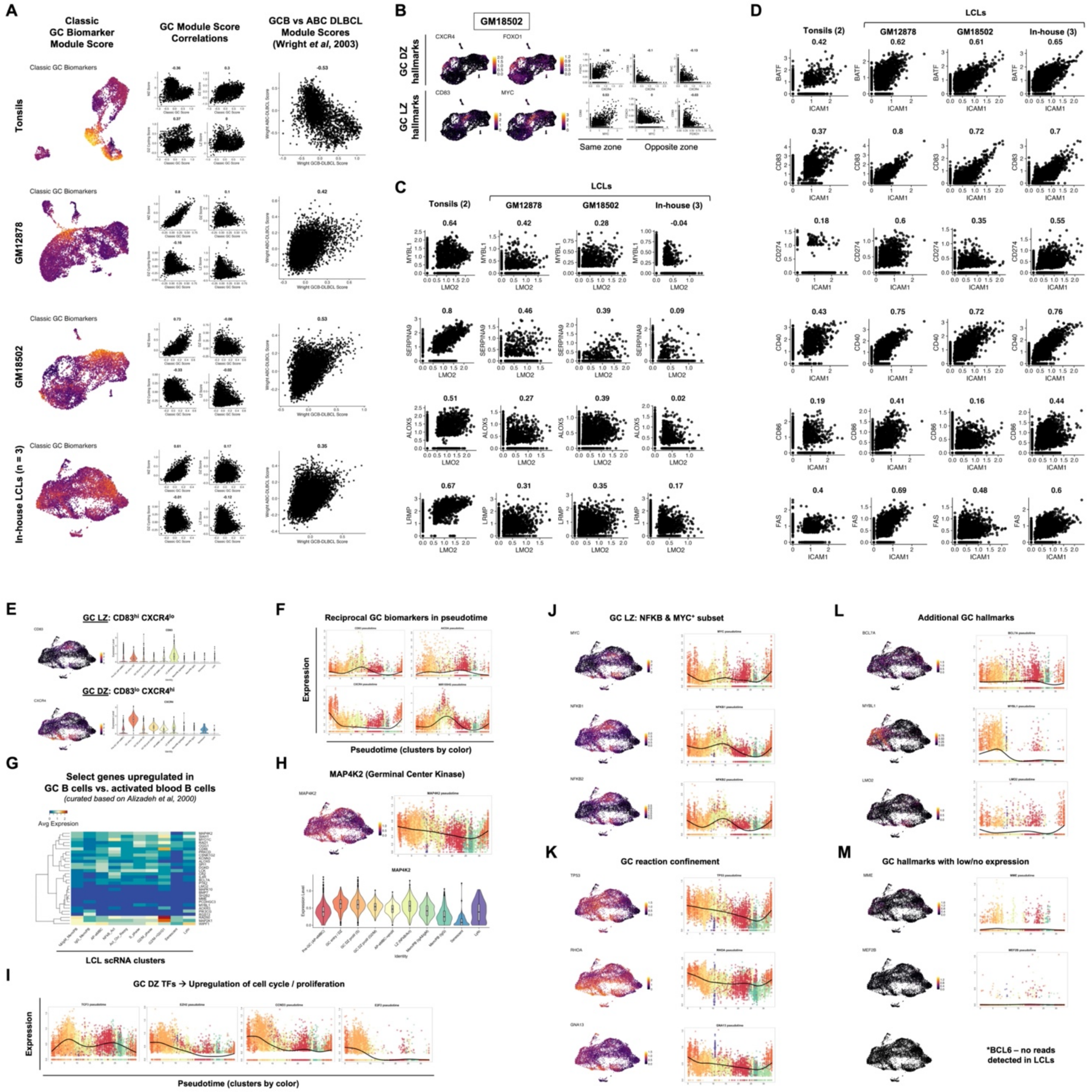

Supporting Figure 3. GC B cell and DLBCL gene expression in LCLs and tonsils. (A) GC B cell biomarker scoring in tonsil and LCL scRNA-seq datasets (left column). Gene module correlations between Classic GC, DZ, DZ cycling, and LZ markers (middle column). Correlation between GCB-DLBCL and ABC-DLBCL module scores. [Classic GC module = *BCL6, LMO2, MYBL1, MME, SERPINA9, GCSAM, DGKD, IL4R, SPI1, SH2B2, ALOX5, BCL7A, LCK, OGG1*; DZ module = *AICDA, FOXO1, CXCR4, AURKC, IL2RB*; DZ cycling module = *TCF3, EZH2, CCND3, E2F2, TP53, PLK4, BRCA1*; LZ module = *NFKB1, NFKB2, CD80, CD83, CD86, BCL2A1, EBI3, CD40, CR2, MIR155HG, ACKR3, MYO1C, MYC*]
(B) Co-expression of canonical GC DZ markers (*FOXO1*, *CXCR4*) and GC LZ markers (*MYC*, *CD83*) in cell subsets within GM18502.
(C) Co-expression of *LMO2* and other classic GC B cell markers (*MYBL1, SERPINA9, ALOX5, LRMP*) across tonsil and LCL scRNA-seq datasets.

(D) Co-expression of *ICAM1* with LZ GC B cell markers (*BATF, CD40, CD83, CD86, CD274, FAS*) across tonsil and LCL scRNA-seq datasets.
(E) Anti-correlated expression of the GC LZ marker *CD83* and GC DZ marker *CXCR4* across 3 in-house LCL scRNA-seq datasets.
(F) Pseudotime-resolved anticorrelated expression of DZ (*AICDA*, *CXCR4*) versus LZ (*CD83*, *MIR155HG*) genes. Cells are ordered in pseudotime and colored by high-resolution clusters as shown in Figure 3D-E.
(G) Cluster-resolved average expression of genes upregulated in GC B cells relative to activated blood B cells (curated from Alizadeh *et al*, 2000).
(H) Pseudotime- and cluster-resolved expression of MAP4K2 (Germinal Center Kinase, identified from Alizadeh *et al*, 2000).
(I) Expression of GC DZ-associated transcriptional regulators (*TCF3*, *EZH2*) and their transcriptional targets upregulating cell proliferation (*CCND3*, *E2F2*) in pseudotime.
(J) *MYC* and *NFKB* family transcription factor expression in pseudotime peaks in GC LZ-like cells.
(K) Genes with known roles in constraining the GC reaction exhibit elevated expression in DZ and DZ cycling cells.
(L) Expression of GC B cell biomarker genes *BCL7A, MYBL1, and LMO2* across in-house LCLs.
(M) Expression of the GC B cell biomarker *MME* and the transcriptional regulators *MEF2B* and *BCL6* is limited or absent from EBV-induced GC-like properties in LCLs.

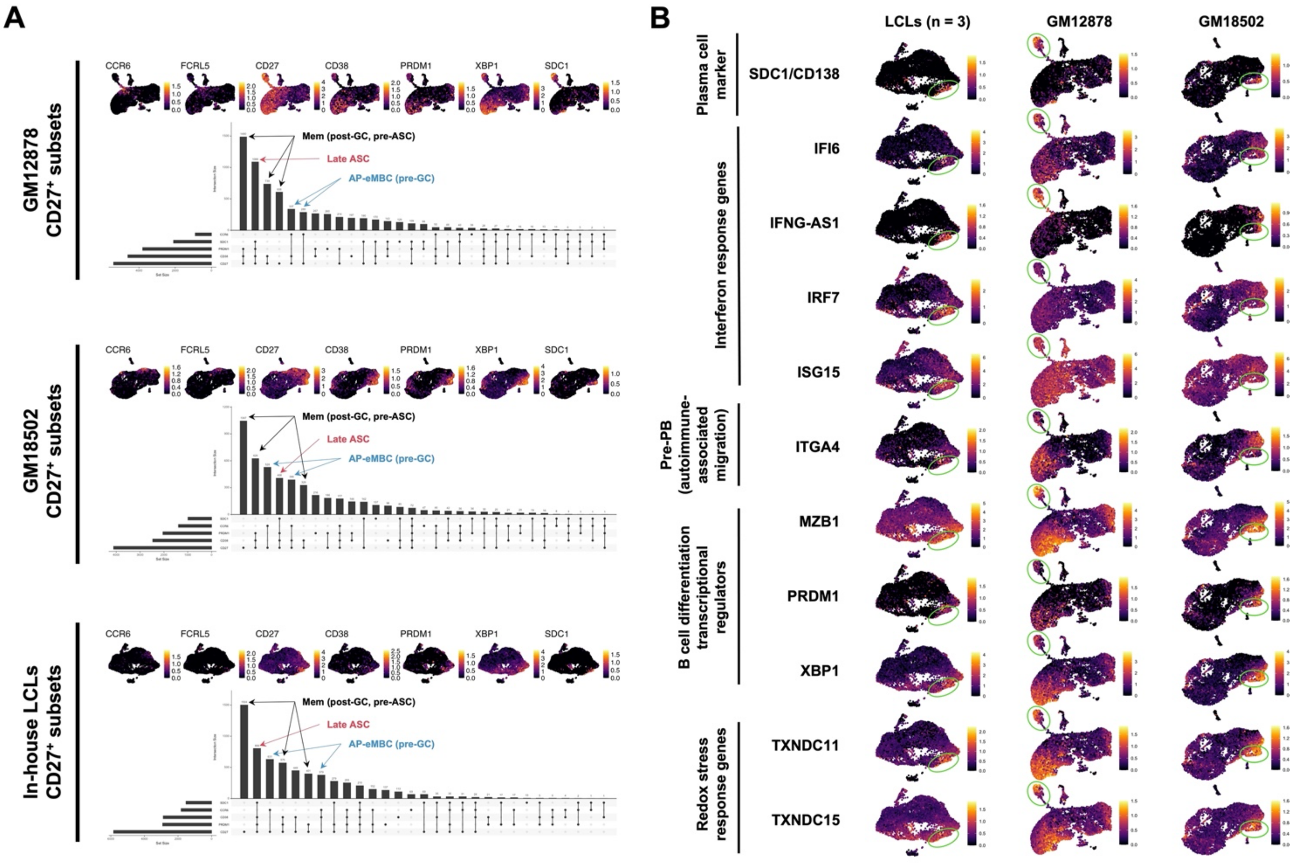

Supporting Figure 4. Distinct *CD27*$^+$ subsets and key marker genes in plasma cells across LCLs.
(A) UMAP and upset plots of *CD27* co-expression with markers of pre-GC early activation (*CCR6*), post-GC memory B cells / pre-plasmablasts (*CD38, PRDM1*), and late ASCs (*SDC1*) across LCLs. Upset plots depict cells with co-expression at levels greater than or equal to the 25th percentile for each gene's respective distribution across the given dataset.
(B) Top markers within late plasma cells (ASCs). Data are presented for three LCLs generated in-house (left column) and commercially available LCLs (GM12878, middle column; GM18502, right column) originally reported by Osorio *et al*[1].

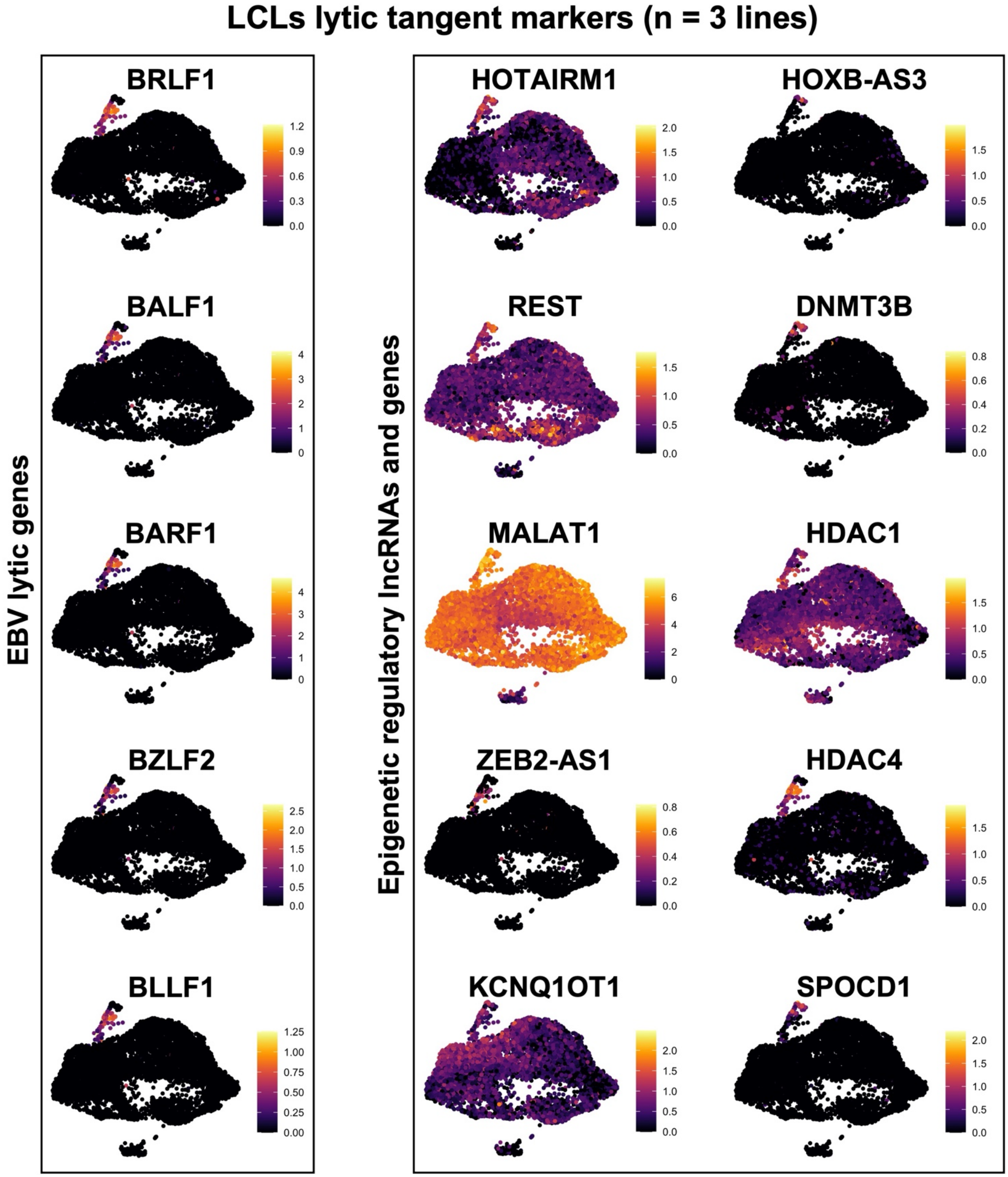

Supporting Figure 5. Key marker genes in lytic cells across LCLs. Data from differentially expressed genes of interest are presented for three LCLs generated in-house.

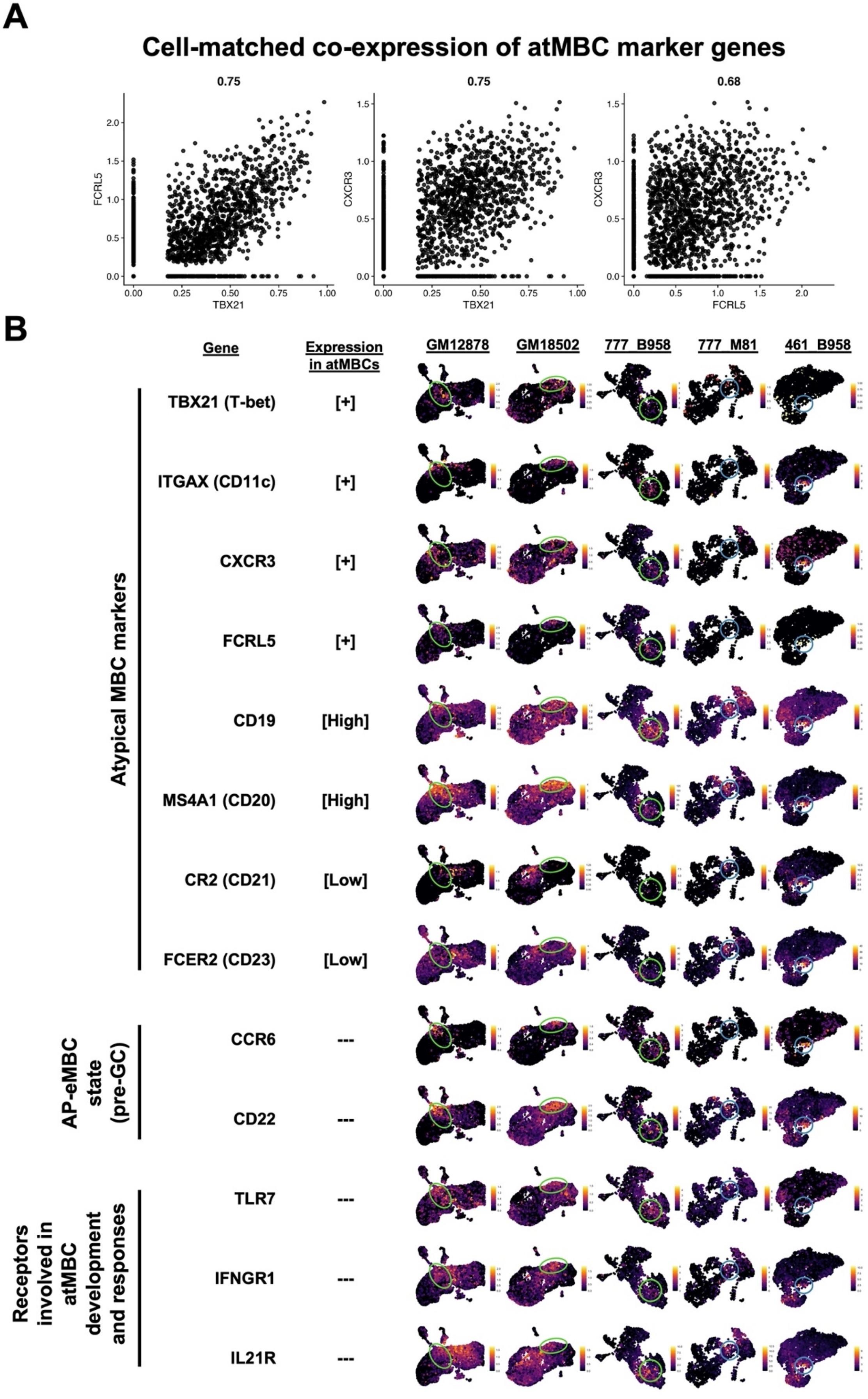
A
Cell-matched co-expression of atMBC marker genes
0.75
0.75
0.68
FCRL5
TBX21
CXCR3
TBX21
CXCR3
FCRL5
B
Gene
Expression in atMBCs
GM12878
GM18502
777_B958
777_M81
461_B958
Atypical MBC markers
TBX21 (T-bet) [+]
ITGAX (CD11c) [+]
CXCR3 [+]
FCRL5 [+]
CD19 [High]
MS4A1 (CD20) [High]
CR2 (CD21) [Low]
FCER2 (CD23) [Low]
AP-eMBC state (pre-GC)
CCR6 ---
CD22 ---
Receptors involved in atMBC development and responses
TLR7 ---
IFNGR1 ---
IL21R ---

Supporting Figure 6. Expression signature of atMBCs within LCLs.
(A) Pearson R correlations for single-cell co-expression of the atMBC markers *TBX21*, *FCRL5*, and *CXCR3* in LCLs.
(B) Data from differentially expressed genes of interest are presented for three LCLs generated in-house and commercially available LCLs (GM12878, GM18502) originally reported by Osorio *et al*[1]. Three of five LCL datasets contain cells consistent with atMBCs exhibiting elevated expression of key receptors that mediate atMBC responses to stimuli in addition to AP-eMBC characteristics (identified by green circles). Cells with AP-eMBC characteristics but lacking definitive atMBC gene expression in the remaining two LCLs are also highlighted (blue circles).

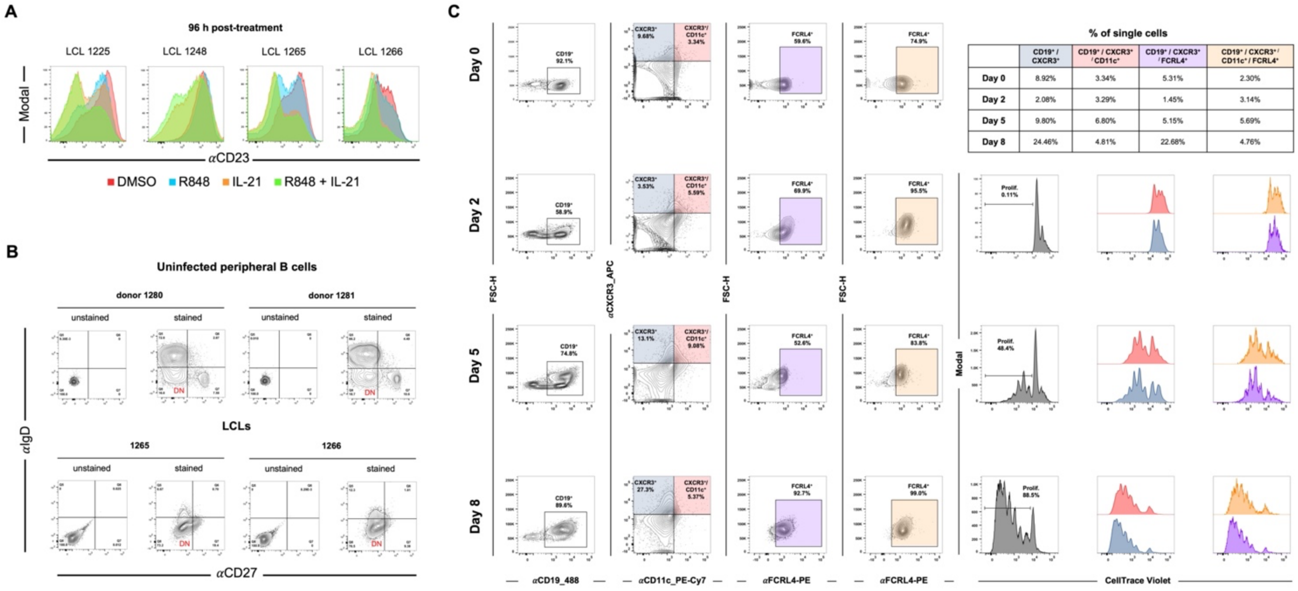

	CD19⁺ / CXCR3⁺	CD19⁺ / CXCR3⁺ / CD11c⁺	CD19⁺ / CXCR3⁺ / FCRL4⁺	CD19⁺ / CXCR3⁺ / CD11c⁺ / FCRL4⁺
Day 0	8.92%	3.34%	5.31%	2.30%
Day 2	2.08%	3.29%	1.45%	3.14%
Day 5	9.80%	6.80%	5.15%	5.69%
Day 8	24.46%	4.81%	22.68%	4.76%

Supporting Figure 7. Additional replicates for FACS studies of EBV-associated autoimmune phenotypes in LCLs and *de novo* infection.
(A) FACS replicates for CD23 (FCER2) downregulation upon stimulation with R848 and/or IL-21.
(B) FACS staining of IgD and CD27 in uninfected peripheral B cells to identify putative double negative (DN) B cell frequencies. Samples were prepared via B cell enrichment (negative isolation) from PBCMs. Data are presented for two donors.
(C) Biological replicate for cell proliferation and CXCR3, CD11c, FCRL4 staining in *de novo* infection experiments.
